# The effect of flanking bases on direct and triplet sensitized cyclobutane pyrimidine dimer formation in DNA depends on the dipyrimidine, wavelength and the photosensitizer

**DOI:** 10.1093/nar/gkab214

**Published:** 2021-04-13

**Authors:** Chen Lu, Natalia Eugenia Gutierrez-Bayona, John-Stephen Taylor

**Affiliations:** Department of Chemistry, Washington University, One Brookings Dr., St. Louis, MO 63130, USA; Department of Chemistry, Washington University, One Brookings Dr., St. Louis, MO 63130, USA; Department of Chemistry, Washington University, One Brookings Dr., St. Louis, MO 63130, USA

## Abstract

Cyclobutane pyrimidine dimers (CPDs) are the major products of DNA produced by direct absorption of UV light, and result in C to T mutations linked to human skin cancers. Most recently a new pathway to CPDs in melanocytes has been discovered that has been proposed to arise from a chemisensitized pathway involving a triplet sensitizer that increases mutagenesis by increasing the percentage of C-containing CPDs. To investigate how triplet sensitization may differ from direct UV irradiation, CPD formation was quantified in a 129-mer DNA designed to contain all 64 possible NYYN sequences. CPD formation with UVB light varied about 2-fold between dipyrimidines and 12-fold with flanking sequence and was most frequent at YYYR and least frequent for GYYN sites in accord with a charge transfer quenching mechanism. In contrast, photosensitized CPD formation greatly favored TT over C-containing sites, more so for norfloxacin (NFX) than acetone, in accord with their differing triplet energies. While the sequence dependence for photosensitized TT CPD formation was similar to UVB light, there were significant differences, especially between NFX and acetone that could be largely explained by the ability of NFX to intercalate into DNA.

## Graphical Abstract

**Graphical Abstract:**
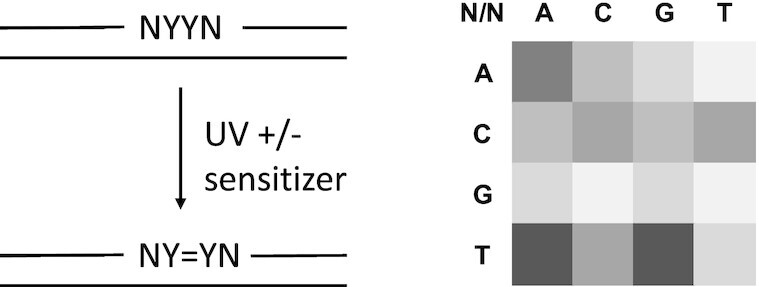
By using a sequence designed to contain all possible NYYN sites, the effect of the dipyrimidine and its flanking sequence, as well as wavelength, and sensitizer on the relative frequency of cyclobutane pyrimidine dimer formation was revealed.

## INTRODUCTION

**Figure 1.**
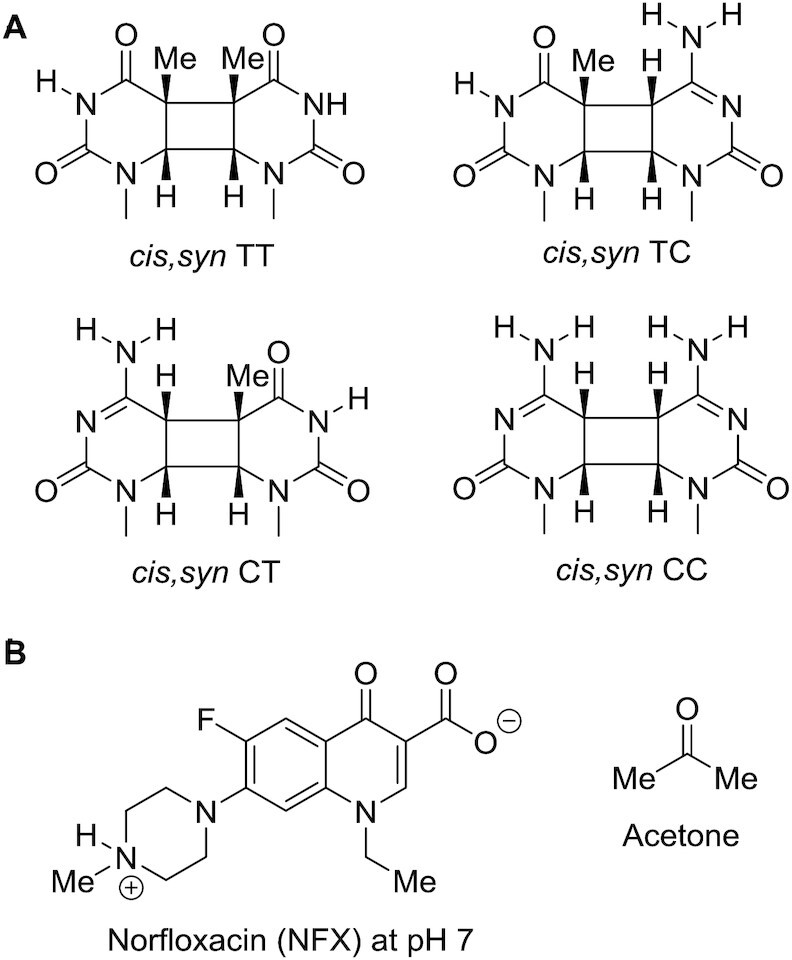
Structures of CPDs and photosensitizers. (**A**) Structures of the *cis-syn* TT, TC, CT and CC CPD’s prior to deamination. Extended incubation at pH 7 will convert the C’s in CPDs to U’s. (**B**) Structure of the triplet sensitizers norfloxacin and acetone.

Cyclobutane pyrimidine dimers (CPDs) (Figure 1A) are the major DNA photoproducts induced by sunlight and are responsible for the majority of C to T and CC to TT mutations leading to skin cancer (1–3). Because CPDs do not distort DNA structure very much, they are repaired with a half life of about 24 h in human cells, which is sufficient time for a C or a 5-methylC (mC) at CpG sites in a CPD to deaminate to a U (or T) (4). Once deaminated, translesion synthesis past U (or T)-containing CPDs by polymerase eta results in the characteristic C to T and CC to TT signature mutations of UV light at dipyrimidine sites (5) as can be inferred from translesion studies of mCT and TmC CPDs and their deamination products (6,7) as well as a TU CPD resulting from deamination of a TC CPD (8). A recent study showed that CPDs can also be generated by a dark pathway following UVA and UVB irradiation that has been proposed to occur through a chemisensitization mechanism involving as yet unknown intermediates (9,10). This pathway is proposed to involve formation of a triplet excited state during the decomposition of a high energy intermediate produced by peroxynitrite that can transfer its energy to DNA to form the CPDs through triplet-triplet energy transfer in the same way that a photosensitizer works. The frequency of CPD formation by direct irradiation has been found to depend on the wavelength and dose, the dipyrimidine site and its flanking sequence, DNA conformation, and protein binding (2,3,11–13). The effect of the triplet sensitizer and flanking sequence on triplet sensitized CPD formation, however, is not as well studied or understood. To better understand these effects, and how they may contribute differently to mutations than by direct irradiation one has to understand the mechanisms of CPD formation by these two modalities in native DNA.

**Figure 2.**
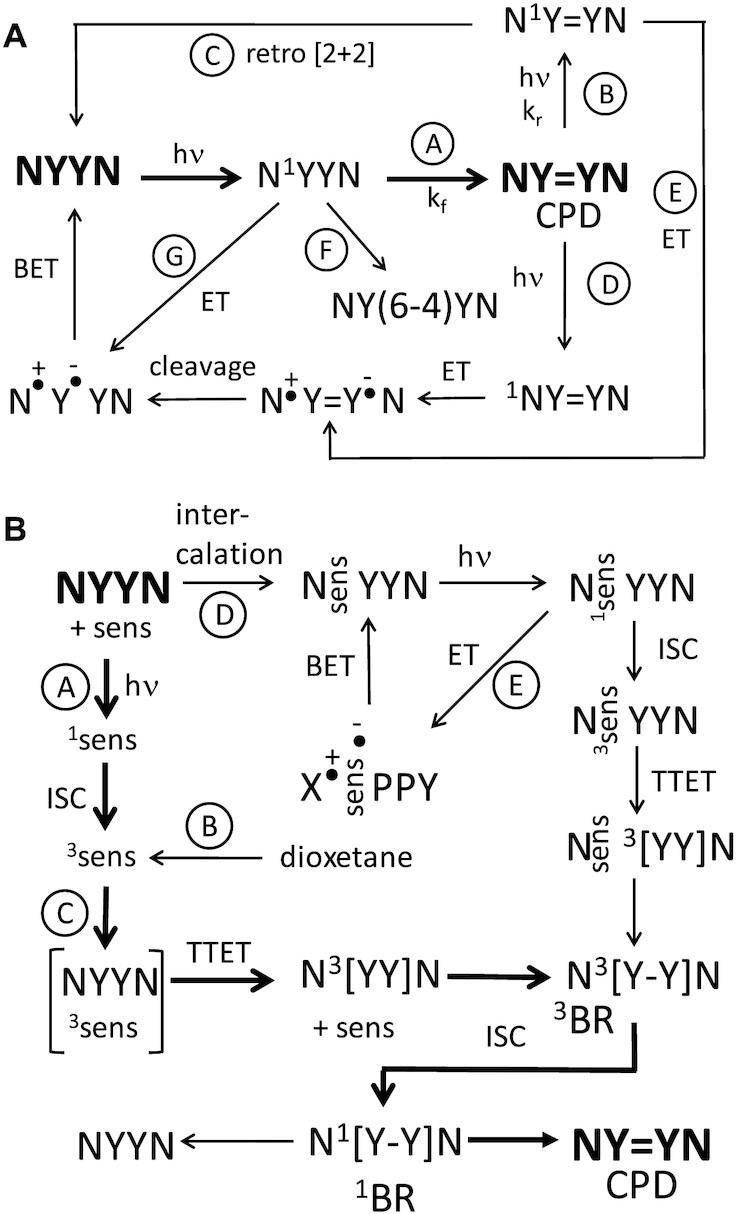
Schemes for direct and photosensitized CPD formation. To simplify the illustration, excited states are shown as localized, but may be delocalized in DNA. (**A**) Scheme for direct excitation in which the singlet state of a pyrimidine (Y) undergoes a [2+2] cycloaddition (indicated by ‘=’) with an adjacent pyrimidine (path A). CPDs are not photostable at 254 nm and can reverse back to the parent dinucleotides via direct excitation of the CPD (path B) followed by a retro [2+2] reaction (path C). It has also been proposed that an excited flanking base could transfer an electron to the CPD to give a charge transfer species that catalyzes the reversal (Path D). Alternatively, there could be an electron transfer from a flanking base to an excited CPD to give a charge transfer intermediate (path E). The rate of CPD formation could also be diminished by competitive formation of a (6-4) photoproduct from the excited singlet state (path F). The excited singlet state could also be deactivated by electron transfer from a flanking base pair (path G) followed by back electron transfer (BET) to the ground state. (**B**) Scheme for the triplet sensitized formation of CPDs involving TTET. In photosensitization, the sensitizer is first excited to the singlet state (^1^sens) followed by intersystem crossing to the triplet state (^3^sens) (Path A). In chemisensitization, the triplet sensitizer is produced by thermal decomposition of a high energy intermediate such as a dioxetane (Path B). The triplet sensitizer then transfers its energy to a dipyrimidine (YY) by TTET (Path C). The triplet dipyrimidine then dimerizes to form a C6–C6 bonded intermediate triplet biradical (^3^BR) that undergoes intersystem crossing (ISC) to the singlet (^1^BR) followed by C5–C5 bond formation to yield the cyclobutane dimer. TTET could occur through either a collision complex (path A) or through an intercalated intermediate (Path D). Sensitizers may also be quenched by electron transfer from a stacked base to give a lower energy charge transfer complex (Path E). Sequence context effects may arise from a combination of binding affinity, pi stacking geometry, and competitive deactivation of the singlet excited state by electron transfer (Path E).

UVC (200–290 nm), UVB (290–320 nm) and UVA (320–400 nm) give rise to CPDs via direct excitation of DNA (2,12,14). In native B form DNA CPDs with the *cis,syn* stereochemistry form via a [2+2] photocycloaddition between the C5–C6 double bonds of two stacked pyrimidine bases (Figure 1A). Ultrafast time-resolved experiments showed that CPDs are formed within less than 1ps upon exposure of single-stranded (dT)_18_ to UVB light (15). The reaction is proposed to proceed via absorption of light to form a localized or delocalized singlet state (Path A, Figure 2A), with minor contribution from triplet states (11,12,16,17). UVC light, and in particular 254 nm light used in most studies, can also photochemically reverse the initially formed CPDs by a retro [2+2] reaction (Path B+C, Figure 2A). As a result, CPD distribution has been found to depend on both the time and wavelength of irradiation, as well as the flanking sequence (18,19). At short irradiation times, CPD formation is in the presteady state regime dominated by the forward rate constant, k_f_, whereas at long irradiation times, the photoproduct distribution approaches a steady state value corresponding to *k*_f_/(*k*_f_ + *k*_r_) (19). It has also been proposed that CPDs might be photoreversed by electron transfer from a photo-excited flanking base, principally G, to give an intermediate radical ion pair that splits the cyclobutane ring (Path D, Figure 2A) (20). Alternatively, the excited state of the CPD could abstract an electron from a flanking base to give the same radical ion pair (Path E, Figure 2A). Subsequent studies with trinucleotides found no evidence for electron transfer reversal mechanisms, and concluded that reversal was occurring via direct excitation of the CPD (21). Because the absorbance of CPDs rapidly diminishes above 260 nm, photoreversal by direct excitation does not occur by this mechanism under UVB light (21). It is possible, however, that photoinduced electron transfer from a neighboring base (Path D, Figure 2A) might also contribute to photoreversal of CPDs in certain conformations as found for some G-quadruplexes (22,23).

There have been a number of studies on the frequency of CPD formation in native duplex DNA *in vitro* by direct irradiation utilizing a variety of methods. In one early study, UV irradiated DNA containing tritiated thymine or cytosine was degraded with acid to release the dimerized bases as their deaminated products Thy=Thy, Thy=Ura, Ura=Thy and Ura=Ura, which could then be separated by paper chromatography and quantified, except Thy=Ura and Ura=Thy, which are enantiomers (24). That study determined the ratio of TT:TC+CT:CC CPDs to be 62:25:13 when irradiated at 265 nm and 72:18:9 at 280 nm following corrections for dinucleotide frequency. A more modern approach uses enzymes to degrade the DNA to photoproduct-containing dinucleotides T=T, T=dU, dU=T and dU=dU which are then separated and quantified by high performance liquid chromatography coupled with electrospray tandem mass spectrometry (HPLC-MS/MS) (25,26). In one early study by this method, the pre-steady state ratio of CPD formation at TT, TC, CT and CC for 254 nm-irradiated calf thymus DNA was 42:41:15:02 (after correction for dinucleotide frequency), which changed to 35:39:19:7 with broad band UVB irradiation (280–370 nm) (25). Analysis of data from a later study of CPD induction by broadband UVB light in genomic DNA with varying GC content (27) gave an average ratio of 36 ± 4: 32 ± 3: 20 ± 5: 13 ± 2 for TT:TC:CT:CC CPD formation.

CPD formation has also been studied by quantifying cleavage sites by CPD specific pyrimidine dimer glycosylases, such as *M. luteus* UV specific endonuclease (28) and T4 pyrimidine dimer glycosylase (29) using end-labeled DNA, ligation mediated PCR or NextGen sequencing. One early study of only 12 of the 64 possible NYYN sites gave a ratio of 68:13:16:03 for 1 kJ/m^2^ UVC and 52:19:21:07 for 8 kJ/m^2^ UVB (30). Quantification of data from a figure in a recent Next Generation sequencing study of a much lower dose of 40 J/m^2^ UVC irradiated human 293T DNA (31) gives a ratio of 32:32:14:21 when corrected for dinucleotide frequency. The differences in CPD ratios between the various studies is likely due to the differences in the doses of light used, sequence coverage, and methods of analysis. The increase in CPD formation at C-containing sites with increasing wavelength that was reported in the early literature can be explained by the use of larger doses of UVC light and a decrease in the photoreversal efficiency with increasing wavelength, which is more efficient for C-containing CPDs (18,19,21). Some of the increase at TC sites may also be due to a decrease in the competitive formation of TC (6-4) photoproducts (Path F, Figure 2A), which has been attributed to result from a charge transfer state whose quantum yield relative to CPDs decreases with increasing wavelength (16).

The effect of flanking sequence context on CPD formation has largely been obtained by gel electrophoresis of end-labeled DNA that has been cleaved with CPD specific endonucleases. Studies have been greatly limited, however, by the DNA substrates used, which have never contained all possible 64 NYYN sequence contexts. In addition, few studies using end-labeled DNA fragments have explicitly taken into account Poisson statistics in their analysis, without which cleavage bands near the labeled end will appear to have higher frequency (18,19). Early studies, using Poisson analysis (18,32–34), along with another study not documenting such an analysis (30), generally concluded that CPD formation by UVC light was more favorable at the 3′-end of a pyrimidine tract and was inhibited by a 5′-G. It was also shown that TT CPDs achieved higher steady state values with UVC than C-containing CPDs (18). A more recent study of CPD formation by 254 nm light at 8 out of 16 possible NTTN sites and 6 out of 16 NTCN sites found that TT flanked by purines had the lowest *k*_r_ and *k*_f_ rates, and that C-flanked sites had the highest rates, while the lowest steady state frequencies were for TT sites flanked by a 5′-G (19). It was also found that *k*_f_ for TC sites was 30–100% that of TT sites, but that *k*_r_ was much higher, thereby explaining the low frequency of C-containing CPDs with UVC light at higher doses. Flanking purines have been shown to increasingly inhibit CPD formation with UVB in the order inosine < adenine < guanine, which was attributed to quenching of the pyrimidine excited state by electron transfer from the flanking base according to oxidation potential (Path G, Figure 2A) (4). It was also proposed that a 5′-G suppressed CPD formation more than a 3′-G due to better pi stacking of the G with the 5′-pyrimidine. Subsequent experimental and theoretical studies supported the electron transfer quenching hypothesis (35–37).

While the triplet state is a minor contributor to CPD formation upon direct excitation with light, it can be directly produced by irradiation of DNA in the presence of triplet sensitizers (38) or by chemiexcitation with dioxetanes (39,40). There has been renewed interest in chemisensitized formation of CPDs following the discovery that CPDs are produced in the dark following UVB and UVA irradiation of melanocytes (9). The pathway has been proposed to involve the formation of triplet excited carbonyl compounds from thermal decomposition of high energy dioxetanes produced via the action of peroxynitrite on melanin metabolites (10). In photosensitized processes (Figure 2B), an organic molecule, the sensitizer (sens), absorbs the UV light and is excited to a singlet state (^1^sens) (Path A) that intersystem crosses to the triplet state (^3^sens). In a chemisensitized process the triplet sensitizer is produced as a result of the thermally induced crossing of the ground state of a high energy molecule, like a dioxetane, to the triplet excited state of a decomposition product (Path B). The triplet excited molecule produced by either process then transfers its energy to the ground state of DNA by triplet triplet energy transfer, or TTET, which involves double electron exchange termed the Dexter mechanism (41–43). This transfer can occur via a collision complex (Path C), or through a bound state, such as intercalated intermediate (Path D). The efficiency of the TTET process depends on the difference in triplet energy of photosensitizer and DNA bases, the coupling interaction between them, and the extent to which the photosensitizer binds to the DNA. Various ketones (44,45) and fluoroquinolones (46–48) with triplet states above those of pyrimidines are known to be good sensitizers of CPD formation (49). A triplet excited pyrimidine can then form a single bond from C6 to C6 of the adjacent pyrimidine leading to a triplet biradical intermediate (^3^BR). The triplet biradical can then intersystem cross to the singlet state (^1^BR) and either go on to the CPD or reverse back to the original dipyrimidine (50,51).

Acetone (Figure 1B), which has a triplet energy of 337 kJ/mol (52) that is above that of both cytosine and thymine, produces 91% CPDs at TT sites in *Escherichia coli* DNA (after correcting for dipyrimidine frequencies) (53). Lamola found a somewhat lower frequency of 86% for CPDs at TT sites in *E. coli* (after correcting for dipyrimidine frequencies) when the acetone triplet state was produced via thermal decomposition of tri-methyl dioxetane at 70 °C (39). It was also found that acetophenone, which has a lower triplet energy of about 317 kJ/mol (54), increased the fraction of TT CPDs to 94% (53). In an early HPLC–MS/MS study of acetophenone sensitized CPD formation in calf thymus DNA, CPDs were found to form with a very different ratio of 55:22:23 at TT:TC:CT (after correcting for base frequency) (55). In a later study, the ratio was reported to be an average of 80:7:13:1 for calf thymus and *Micrococcus luteus* DNA (corrected for dipyrimidine frequencies) (41). When DNA is irradiated with norfloxacin (NFX) (Figure 1B), which has the lowest triplet energy known to sensitize CPD formation (269 kJ/mol) (49), the average ratio for TT:TC:CT:CC was reported to be 87:5:7:1 (41). Although formation of CPDs by TTET has been studied for many years, the effect of flanking sequence on CPD formation has never been studied in any detail. One early study with acetone and 313 nm light found that the majority of CPDs formed at TT sites, and that CPD formation decreased in the order GTTG > GTTA > ACTG > ATCG > ACCA for the five sites studied (56). A study with pyridopsoralen photosensitized CPD formation found hotspots at CTTA > TTTA which were attributed to either selective binding or better pi orbital overlap of the psoralen with the thymines (57).

While collectively, CPD formation by direct irradiation may have been studied in all flanking sequence contexts *in vitro*, it is very difficult to impossible to correlate the results between studies because of the lack of internal standards and uniformity in the experimental conditions of irradiation and methods of analysis. As far as triplet sensitized CPD formation, there is very little sequence context data. To gain a more complete picture of the effect of flanking sequence on CPD formation in native DNA, we have determined the relative yields of CPD formation by direct and sensitized pathways in a single sequence that contains one instance of each of all possible 64 NYYN sequences using a CPD-specific enzymatic assay. We find that a 5′-flanking G has a very strong inhibitory effect on UVB and UVC induced CPD formation at all dipyrimidine sites, and that the majority of CPDs occur at YYYR sites, and in particular TYYA. We also found that while the sequence context effects on triplet sensitized CPD formation by both acetone and NFX were similar to those for UV light, they differed significantly between themselves, most likely due to the ability of NFX to intercalate into DNA. These results indicate that photosensitizers may produce unique patterns of CPD formation that could be used as signatures for identifying the chemisensitizers involved in the recently discovered dark pathway to CPDs. The sequence context data obtained may also be useful for guiding physical and theoretical studies of CPD formation by direct and sensitized pathways that may lead to a better understanding of the excited states involved and how they are influenced by flanking sequences.

## EXPERIMENTAL

### Materials

Oligodeoxynucleotides (ODNs) were purchased from Integrated DNA technologies (IDT), [γ-^32^P] ATP from Perkin Elmer, and T4-pdg (pyrimidine dimer glycosylase, previously known as T4 endonuclease V) was prepared from a clone provided by Stephen Lloyd of the Oregon Institute of Occupational Health Sciences.

### Python code for generating sequence libraries

A python script was written to randomly merge one instance of each of all 64 NYYN sites by overlapping identical ends of the tetramers. In addition, the sequences were limited to having at most four consecutive pyrimidine bases. Examples of other 129-mer sequences with all 64 possible sites are shown in Supplementary Figure S1.

### Synthesis of the 129-mer containing plasmid

A 171-mer DNA containing the desired 129-mer DNA sequence flanked by EcoRI sites was synthesized by template-directed ligation of a 48-mer, 42-mer, 41-mer and 40-mer using partially complementary 39-mer, 38-mer, 34-mer scaffolds (Supplementary Figure S2). The ODNs were annealed in equal molar ratios by heating to 95°C and slowly cooling to room temperature in 50 mM NaCl. Ligation was then carried out overnight at 23°C with T4 DNA ligase (Promega) in 1 mM adenosine triphosphate (ATP), 30 mM Tris–HCl (pH 7.8 at 25°C), 10 mM MgCl_2_ and 1 mM dithiothreitol (DTT). An aliquot of the ligation reaction was then subjected to 19 cycles of PCR to obtain the 171-mer double-stranded DNA (dsDNA) with 4 μl of 1 unit/μl LongAmp Taq (New England Biolabs), 0.5 μM of forward and reverse primers, and 300 μM of dNTPs, in 100 μl of 1× LongAmp Taq DNA polymerase buffer. The solution was then phenol extracted, ethanol precipitated, and electrophoresed on a 10% native PAGE (10% acrylamide, 0.33% bisacrylamide, 1× TBE) along with two 5′-[^32^P]-labeled portions to serve as markers. The bands corresponding to the 171-mer duplex were excised from the gel, crushed, and shaken in 3 ml of ddH_2_O overnight. The eluted DNA was then isolated by phenol extraction and ethanol precipitation, restricted with EcoRI, and cloned into the EcoRI site of pBlueScript II SK-vector DNA (Agilent). A clone containing the desired 129-mer was verified by DNA sequencing (Supplementary Figure S3). The plasmid was then isolated from the clone using the Promega PureYield plasmid miniprep system.

### Preparation of the 5′-[^32^P]-labeled DNA substrates

**Figure 3.**
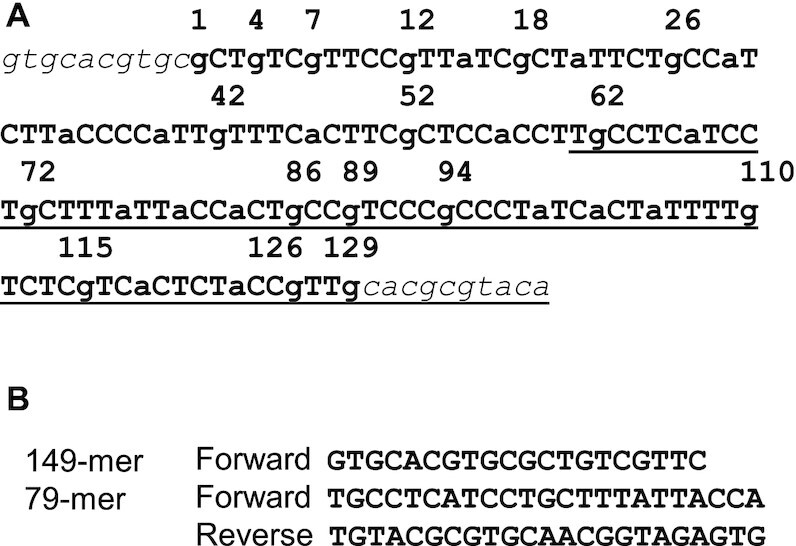
The 149-mer and 79-mer sequences used in this study. (**A**) The sequence in bold is the 129-mer sequence containing one each of all possible NYYN tetrads, where the YY-containing sites are in uppercase, while the 10 nucleotide sequences in italic font are devoid of dipyrimidine sites and used to minimize end effects on CPD formation in the central 129-mer sequence. The underlined sequence corresponds to the 79-mer sequence used to better resolve the bands at the 3′-end of the 129-mer sequence. Numbering of the nucleotides is based on their position in the 129-mer sequence (as shown for the G’s). (**B**) Primer pairs used to produce the 5′-[^32^P]-labeled 149-mer and 79-mers by PCR.

Radiolabeled 149-mer or 79-mer duplex DNA was prepared by PCR amplification from 30 ng of plasmid using 4 μl of 1 unit/μl LongAmp Taq LongAmp Taq DNA polymerase, 0.5 μM 5-[^32^P]-labeled forward primer (Figure 3B), 0.5 μM reverse primer, and 300 μM dNTPs in 100 μl of 1× LongAmp Taq DNA polymerase buffer, and purified by native PAGE.

### UV irradiation

Irradiations were carried out on the 5′- [^32^P]-labeled 149-mer or 79-mer DNA substrates and the indicated amount of photosensitizer (if used) in 20 μl of T4-pdg buffer (50 mM NaCl, 5 mM EDTA, 10 mM Tris–HCl buffer, pH 7.5 in a 1.5 ml polyethylene microfuge tube lying on its side in ice water. The UVC light source consisted of two UVC tubes (XX-15F, Spectroline) delivering ∼8.5 mW/cm^2^ of 254 nm light at 1 cm, corresponding to 85 J/m^2^-s as determined by uridine actinometry in a quartz cuvette (58) and ∼65 J/m^2^-s in the microfuge tube. Broadband UVB (BB UVB, 275–400 nm, centered at 312 nm) irradiation was carried out with two UVB tubes (XX-15B, Spectroline) delivering ∼5.5 mW/cm^2^ at 1 cm (55 J/m^2^-s) using a light meter. Filtered broadband UVB (filtBB UVB, 300–400 nm centered at 330 nm) was carried out by filtering BB UVB through a plate of Pyrex glass and was 2.2 mW/cm^2^ or 22 J/m^2^-s. Narrowband UVB (NB UVB, 310–320 nm centered at 311 nm) irradiation was carried out with a 311 nm UVB Lamp (PLS-9W/01/2P, Philips) delivering 4 mW/cm^2^ at 1 cm or 40 J/m^2^ -s. UVA irradiation (320–410 nm centered at 360 nm) was carried out with a UVA lamp (PL-L 36W UVA, Philips) delivering approximately 15 mW/cm^2^ at 1 cm or 9 kJ/m^2^ -min. The emission spectra and relative intensity of the light sources were obtained with an Ocean Optics spectrometer (Supplementary Figure S4). The UV exposure times ranged between 8 s and 30 min, depending on the experiment and light source, and were adjusted so as to leave no less than 60% uncut DNA following T4 pdg treatment. For 254 nm the time was 8 s corresponding to 520 J/m^2^, for BB UVB the time was 30 s or 1.6 kJ/m^2^, for NB UVB for 10 min or 24 kJ/m^2^, filtBB UVB 30 min or 40 kJ/m^2^. A time study was also carried out with the UVC light to determine the extent to which photoreversal might be contributing to the observed CPD frequencies (Supplementary Figures S5 and S6). Photosensitization with acetone was carried out in 20% acetone in water with 30 s of NB UVB (1.2 kJ/m^2^), whereas photosensitization with 300 μM NFX was carried out with 30 min of UVA (270 kJ/m^2^). For photosensitized reactions, the samples were deaerated by bubbling the samples with nitrogen gas and then capped. All irradiations and analyses for determining photoproduct frequencies were performed in triplicate.

### Mapping of CPD formation by T4-pdg Assay

After UV irradiation, each sample was incubated with 1 μg of T4-pdg (3.2 μM final concentration) for 30 min at 37°C followed by 1 M piperidine at 90°C for 5 min to ensure complete elimination of the sugar ring. The samples were then vacuum dried in a Savant Speedvac, and then vacuum dried twice from ddH_2_O. The dried samples were redissolved in 20 μl of formamide-dye and loaded onto a 0.4 mm 10% denaturing PAGE. As a control, an unirradiated sample was treated in the same way. In addition to the control and UV samples, Maxam and Gilbert G-sequencing reactions were carried out on the 79-mer and 149-mer DNA according to standard procedures (59). The 149-mer samples were loaded at two or three separate times to obtain sufficient resolution for band integration. To demonstrate that the T4 pdg was not biased towards a particular dipyrimidine CPD, and that the 5 min 90 °C treatment did not induce significant cleavage at any (6-4) and Dewar photoproducts, the percent cleavage of the 79-mer and 149-mer fragments with and without prior exposure to 15 s of BB UVB was examined as a function of a 30 min incubation with 0, 0.16, 0.32, 1.6 and 3.2 μM T4 pdg (Supplementary Figures S7 and S8). To demonstrate that the 5 min 90 °C piperidine treatment did not induce significant cleavage of DNA irradiated with UVC, the 79-mer sequence was irradiated with 10 s of 254 nm with and without subsequent irradiation with 30 min of UVB, and with and without T4 pdg followed by 5 min 90°C piperidine (Supplementary Figure S9).

### Quantification and data analysis

The bands in the gels were quantified by the volume rectangle tool in the Biorad Quantity One software. For the direct irradiation experiments, bands were well separated from positions 1 to 52 of the 149-mer for the samples loaded last on the gel, from positions 72 to 129 of the 79-mer for those loaded second, and from positions 42 to 86 of the 149-mer for those loaded first. A number of well resolved bands that overlapped between the three lanes were used to normalize the data between lanes. CPD frequencies were then calculated for each substrate by a previously described method that takes into account Poisson statistics (18,19,34). In brief, the relative CPD frequency was calculated as the band volume divided by the total volume of all bands to the 3′-side of the band. The relative frequency of the bands in the control lane were calculated in the same way and then subtracted from the CPD band frequencies. The calculated relative frequencies of bands at GTTT-43, TTTC-44, TTCA-45 were then used to normalize the data from the first and third loadings, and bands at GCTT-73, CTTT-74, TTTA-75, ATTA-78, ACCA-81, ACTG-84, GCCG-87 sites were used to normalize the bands between the second and third loadings. After the band areas were normalized for all 64 sites, the relative percentages of the bands were calculated by dividing the normalized areas for each site by the total area for all 64 sites, and then averaged from 3 independent experiments.

The relative frequencies for the CPDs produced by the photosensitized reactions were carried out in the same way as for the direct pathway. Bands at C-containing sites could not be quantified for the NFX-sensitized reactions, due to their low intensity, but could be for some sites in the acetone-sensitized reactions. Bands were well separated from positions 1 to 52 of the 129-mer samples loaded last, from 42 to 110 on the samples loaded second, and from 86 to 129 on the samples loaded first. The relative CPD frequencies were then normalized between loadings 1 and 2 using the overlapping bands at ATTG-40, GTTT-43, TTTC-44, CTTC-49 sites. Similarly, the CPD frequencies for loadings 2 and 3 were normalized using overlapping bands at ACTA-103, ATTT-106, TTTT-107, TTTG-108 sites. After the frequencies were normalized for all 16 TT sites, the relative percentage yields were calculated by dividing the frequency for each site by the total frequency of all 16 bands, and then averaged from three independent experiments.

## RESULTS

### Design and synthesis of the sequence

To find the shortest possible sequence containing all 64 possible sequence contexts of NYYN, a computer algorithm was written to combine individual tetrad sites in random order, allowing sites to overlap. To avoid having multiple adjacent CPD sites that could make gel analysis more difficult, the search was further limited to sequences having a maximum of four consecutive pyrimidine bases. The sequences meeting these criteria were then selected according to length, resulting in a library of nine 129-mer sequences. Inspection of the sequences revealed that the 129-mer sequence resulted from joining the 16 possible RYYR sites with the 16 possible YYYY sites to give a sequence of length 16 × 4 + 16 × 4 + 1 = 129 nucleotides. As a result of this shortening, the sequence does not contain any RY_n_R sequences other than for *n* = 2 and 4 as all the YYYY, RYYY and YYYR tetramers are contained within the RYYYYR sequences.

One of the nine 129-mer sequences generated was then randomly selected for the study (Figure 3A and Supplementary Figure S1). To minimize the end effects on CPD formation and T4-pdg cleavage, we added 10-mers lacking dipyrimidines to both the 5′- and 3′- ends, resulting' in a 149-mer DNA (Figure 3A). To clone the 149-mer into a plasmid, we added EcoRI sites d(GAATTC) to both ends of the sequence, flanked by an additional 6 bp to ensure efficient digestion, to produce a 171-mer. The 171-mer was prepared by ligating four shorter DNA sequences with the help of scaffold sequences (Supplementary Figure S2), followed by PCR-amplification. The PCR product was restricted by EcoRI and cloned into the EcoRI site of a pBlueScript SK- vector. Individual clones were screened by enzyme digestion and verified by DNA sequencing (Supplementary Figure S3).

### Analysis of CPD photoproduct formation

To study CPD formation, a radiolabeled 149-mer was prepared by PCR amplification of the plasmid with a 5′-[^32^P]-labeled forward primer (Figure 3B). A 5′-[^32^P]-labeled 79-mer fragment, containing positions 61–149, was also prepared by PCR-amplification to obtain better resolution of bands at the 3′-end of the sequence. Part of the labeled sample was subjected to the Maxam-Gilbert G sequencing reaction, which produces a 3′-phosphate end following treatment with hot piperidine which is the same type of end produced by the T4 pdg glycosidase and lyase activities on the 5′-pyrimidine of the CPD (60). The remaining part of the sample was irradiated with UV light and then treated with T4-pdg followed by hot piperidine treatment (1 M at 90°C for 5 min) to insure complete degradation to a 3′-phosphate end, which is otherwise very inefficient with T4 pdg (60,61). The UV irradiation time was adjusted to achieve quasi single hit kinetics by insuring that at least 60% of the DNA remained uncut by T4 pdg. The T4 pdg cleavage bands were aligned with the sequence by using the Maxam-Gilbert G bands, and quantified by volume integration, and analyzed by taking into account Poisson statistics as described by others (18,19).

While the T4 pdg assay has been used extensively to quantify CPD formation, we were unable to find a study demonstrating its sequence-independence under the conditions normally used for such studies. Because an early study showed that the *K*_m_ of T4 pdg for a TT CPD containing duplex is about 10 nM, and that *k*_cat_ was about 1 min^−1^ (62), we examined the percent cleavage at a number of CPD sites in the 149-mer by a 30 min incubation with T4 pdg at concentrations that were 16 to 320-fold greater than the *K*_m_ (160 nM to 3.2 μM) (Supplementary Figure S7). The percent cleavage at 20 dipyrimidine sites (7 TT, 5 TC, 3 CT and 5 CC) with different flanking sequences was found to only vary by 5 ± 3% at each site over the range of T4 pdg concentrations indicating that CPD cleavage was complete at all sites under the conditions of the assay (3.2 μM) (Supplementary Figure S8). This result is in accord with the lack of dimer and flanking base-specific contacts observed in the crystal structure of an enzyme substrate complex (63).

Because the assay for CPDs used T4 pdg followed by hot piperidine, the control lane was chosen to be the same. It is also known, however, that hot piperidine cleaves (6–4) photoproducts and their Dewar valence isomers which are also produced by UVC and UVB light (64–66) and could contribute to the band intensities of the CPDs, and that Dewar products are more labile than (6-4) photoproducts (61,66,67). The half-lives for cleavage at TT (6-4) and Dewar products in single stranded 49-mers were 140 min and 3 min, respectively (61), indicating that TT (6-4) photoproducts would not contribute significantly to cleavage by the 5 min treatment used in this study, but Dewar products would. Previous experimental work (25,66) and calculations (68) have shown, however, that Dewar photoproducts are not produced in significant yields at the low doses of UVC and UVB used in this study. This is because Dewar product formation requires successive absorption of two photons, one to form the (6-4) photoproduct and a second to photoisomerize the (6-4) product to the Dewar product, both of which have significantly different wavelength maxima of 260 nm (UVC) vs 320 nm (UVB), respectively, and low quantum yields (68).

Initial experiments showed that irradiation of the 149-mer with 15 s of UVB light prior to 5 min of hot piperidine did not result in any obvious new bands compared to treatment with hot piperidine alone, and only increased the background cleavage by 8%. In comparison, prior treatment of the DNA with 3 μM T4-pdg increased the hot piperidine background by 16% (Supplementary Figure S7). Likewise, irradiation of the 79-mer with 10 s of UVC light did not result in any obvious new bands upon treatment with 5 min of hot piperidine and increased the background cleavage induced by piperidine alone by only 10% (Supplementary Figure S9). Sites of (6-4) and Dewar products could be revealed by 5 min of hot piperidine, however, when the DNA that had been irradiated with 10 s of UVC was subsequently irradiated with 30 min of UVB. It was concluded, therefore, that treatment with 3 μM T4-pdg followed by 5 min of 90°C piperidine serves as an acceptable control lane for quantifying CPD formation.

### CPD formation by UV light

**Figure 4.**
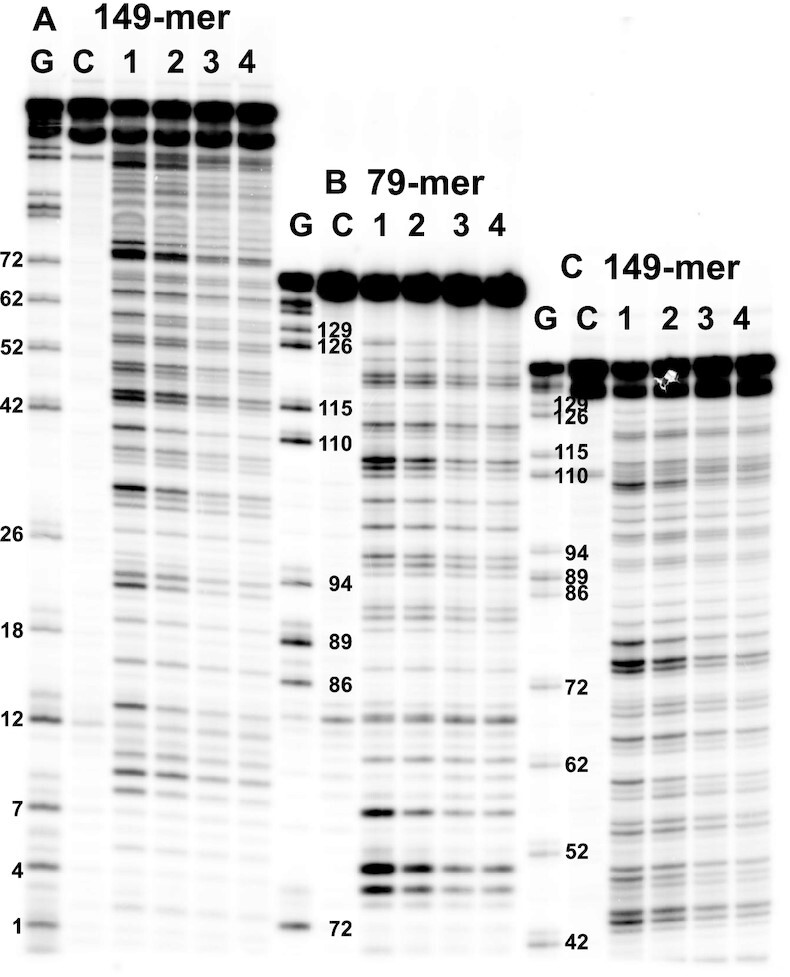
PAGE analysis of CPD formation under different irradiation conditions. The samples were loaded sequentially in order to resolve all 64 cleavage bands on one gel. (**A**) 149-mer sequence loaded last, (**B**) 79-mer sequence loaded second, and (**C**) 149-mer sequence loaded first. Lane G: Maxam Gilbert G ladder, lanes C, 1–4: samples treated with T4-pdg followed by hot piperidine, lane C: control lane without UV treatment, lane 1: UVC, lane 2: BB UVB, lane 3: filtBB UVB, and lane 4: NB UV. The numbered bands correspond to the G’s shown in Figure 2.

**Figure 5.**
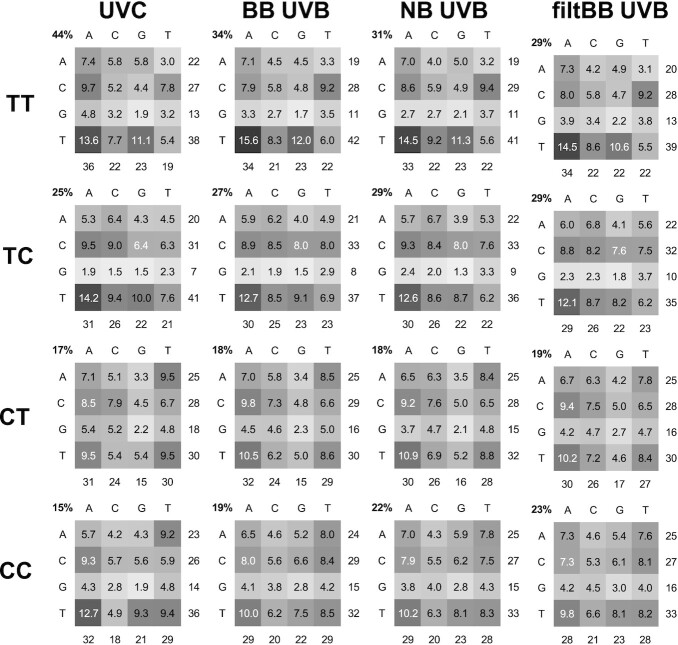
Sequence dependence of CPD formation by UVC and UVB light. Heatmaps of the relative percent of CPD formation at the 16 possible NYYN sites for each of the four possible dipyrimidine sites obtained by quantification of the PAGE gels. The total percent of CPDs formed at each type of dipyrimidine site is given at the top left corner. The rows correspond to the 5′-base and the columns to the 3′-base. The number to the right of a row is the total frequency for the indicated 5′-base, while those below the columns are the total frequency for the indicated 3′-base. See text and Supplementary Figure S4 for the emission spectra of the light sources used. The average of three experiments are shown. The standard deviations are given in Supplementary Figure S10.

The DNA was irradiated with four types of UV light: (1) 254 nm (UVC), (2) broadband UVB from 280 to 400 nm, centered at 312 nm (BB UVB); (3) BB UVB filtered through Pyrex glass to give 300–400 nm, centered at 330 nm (filtBB UVB); and (4) narrowband UVB from 310–320 nm, centered at 311 nm (NB UVB). The relative emission spectra of the UV sources are shown in Supplementary Figure S4. The T4-pdg cleavage bands in the irradiated samples could be resolved well at all 64 different CPD sites on a 10% denaturing PAGE gel using the sequentially loaded 149-mer and 79-mer samples (Figure 4). The bands were best resolved for positions 1–52 for the sample of the 149-mer loaded last, and for positions 42 to 86 for the sample of the 149-mer loaded first. Bands for positions 72–129 were best resolved for the sample of the 79-mer loaded second. The average relative frequency of CPD formation at the different dipyrimidine sites is shown in Figure 5. Histograms for average relative frequencies and their standard deviations for three experiments are given in Supplementary Figure S10.

Because CPDs are easily reversed by UVC light and more so for C-containing sites, which could affect the CPD ratios, a time course experiment was carried out (Supplementary Figure S5 and S6). CPD formation at TT sites and some C-containing sites up to a 30 s exposure to 254 nm light (∼2000 J/m^2^) increased linearly, and hence, appeared to be in the pre-steady state regime. On the other hand, CPD formation at other C-containing sites showed curvature indicating some contribution from photoreversal, though the CPD induction appeared to be linear up to the 8 s time point (520 J/m^2^) that was used for the analysis. Among these sites, TC = CA-55 showed the most curvature which would be expected because the presence of 2 C’s. This is similar to what has been previously observed for these doses (18,19).

Our TT:TC:CT:CC ratio of 44:25:17:15 (TT:TC = 1.76) (Figure 5) for UVC is similar to the ratio of 37:28:18:17 (TT:TC = 1.32) determined by a NextGen analysis of isolated DNA exposed to 1 kJ/m^2^ (calculated from their Figure 3b and corrected for dipyrimidine frequencies) (69) and almost identical to 42:24:16:17 obtained from a NextGen analysis of isolated fibroblast DNA exposed to 200 J/m^2^ (calculated from their Figure 2A and corrected for dipyrimidine frequency) (31). Our ratio differs, however, from 32:32:14:21 (TT:TC = 1) observed in the latter study at a lower dose of 40 J/m^2^, and of 26:24:15:35 (TC:TT = 1) from an LMPCR study at a dose of 30 J/m^2^ (70), suggesting that the high ratio of TT:TC that we observe is due to a contribution from photoreversal. A TT:TC ratio of 1 was also determined by HPLC-MS/MS (42:41:15:02 corrected for dipyrimidine frequency), obtained from linear portion of 0 to 800 J/m^2^ data (25). Compared to our values and those from NextGen sequencing studies, both of which also used T4-pdg to locate CPDs, the value of 2% for the fraction of CC CPDs and 5.5% from a later study (71) determined by the HPLC–MS/MS method seems to be greatly under-estimated. With 1.6 kJ/m^2^ of BB UVB irradiation a ratio of 34:27:18:19 (TT/TC = 1.26) (Figure 5) was obtained which is very similar to the ratio of 36:32:20:13 (TT/TC = 1.13, corrected for dipyrimidine frequency) obtained from an HPLC-MS/MS assay of calf thymus DNA with with 0.8 kJ/m^2^ UVB (27), though we observed a slightly higher fraction of CC CPDs. With 24 kJ/m^2^ of NB UVB and 40 kJ/m^2^ filtBB UVB the ratio becomes ≈30:29:18:23, which is similar to a ratio of 25:29:20:26 obtained by LMPCR analysis of 952 sites in isolated fibroblast DNA with 4 kJ/m^2^ of >290 nm UVB light (72). The ratios for NB and filtBB UVB are very similar to the ratio of 32:32:14:21 at the 40 J/m^2^ dose of UVC observed in the NextGen sequencing study (31), and to the 1:1 TT:TC ratio observed in the HPLC-MS/MS study (25).

### Effect of flanking sequence on CPD formation by UV light

While the average frequency of CPD formation at each of the four dipyrimidines (TT, TC, CT and CC) varied by at most 3-fold between UVC and filtBB UVB, the variation of CPD formation as a function of flanking sequence was much greater (Figure 5). CPD formation was highest at TTTA-75 for all UV sources, and lowest at GCTG-2 for all UVB sources but lowest at GCCG-87 for UVC. For UVC light, CPD formation varied up to 21.4-fold from 6.0% to 0.28%; for BB UVB, 13.3-fold from 5.3% to 0.40%; for NB UVB, 11.6-fold from 4.4% to 0.38%, and for filtBB UVB 8.3-fold from 4.2% to 0.5%. The relative photoproduct frequencies for the 64 different sites (NYYN) correlated fairly well when light sources with similar wavelengths were compared (*R* > 0.94) (Table 1). The worse correlation was between UVC and filtBB UVB (*R* = 0.85), as would be expected because of the absence of any possible contribution from photoreversal. On the other hand the relative photoproduct frequencies correlated equally well for TT and TC across all light sources (*R* ≥ 0.96) while CT and CC showed poorer correlation between UVC and filtBB (≤0.90), reflecting the greater contribution of photoreversal to CT and CC with UVC. For this reason all further discussion of the sequence dependent effects will be for NB UVB.

**Table 1.** Correlation between relative CPD frequencies with different UV light sources and sensitizers as a function of flanking sequence for individual and all dipyrimidine sites

	Site	UVC	BB UVB	NB UVB	filtBB UVB	UVB/acetone	UVA/NFX
UVC	TT	1	0.96	0.96	0.96	0.77	0.74
	TC	1	0.97	0.97	0.97		
	CT	1	0.95	0.93	0.90		
	CC	1	0.91	0.92	0.88		
	YY	1	0.94	0.87	0.85		
BB UVB	TT	0.96	1	0.99	0.99	0.74	0.60
	TC	0.97	1	0.99	0.99		
	CT	0.95	1	0.99	0.98		
	CC	0.91	1	0.98	0.98		
	YY	0.94	1	0.97	0.96		
NB UVB	TT	0.96	0.99	1	0.99	0.72	0.58
	TC	0.97	0.99	1	1.00		
	CT	0.93	0.99	1	0.99		
	CC	0.92	0.98	1	0.98		
	YY	0.87	0.97	1	0.99		
Filt BB	TT	0.96	0.99	0.99	1	0.74	0.60
	TC	0.97	0.99	1.00	1		
	CT	0.90	0.98	0.99	1		
	CC	0.88	0.98	0.98	1		
	YY	0.85	0.96	0.99	1		
UVB/acetone	TT	0.77	0.74	0.72	0.74	1	0.47
UVA/NFX	TT	0.74	0.60	0.58	0.60	0.47	1

### Effect of flanking sequence on CPD formation by NB UVB

**Figure 6.**
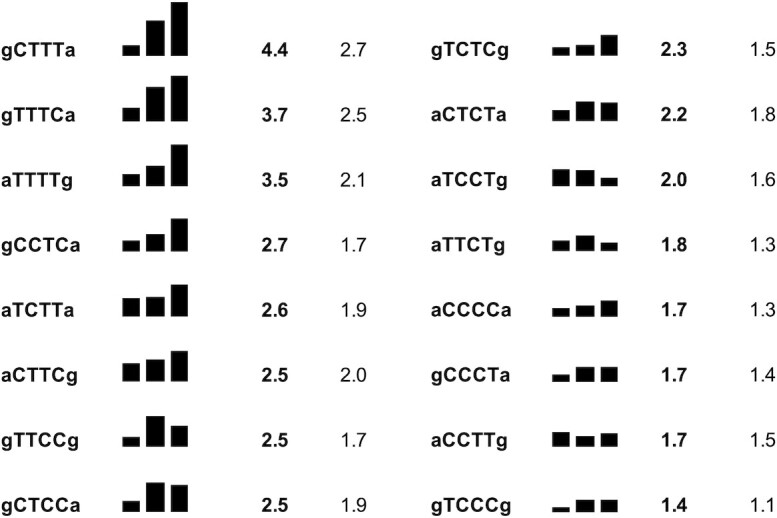
Photoproduct distribution in RYYYYR sites. CPD formation in the 16 RYYYYR sites by NB UVB light are ranked according to the most frequent CPD within the pyrimidine tract whose percentage is given immediately to the right of the sequence followed by the average frequency for all three CPD sites within the tract. The relative frequency of CPD formation within the pyrimidine tracts are shown as a barplot with the same scale. The statistical probability for 1/64 sites is 1.6%.

At the trinucleotide level, CPD formation with NB UVB was most frequent with a 5′-flanking pyrimidine YYY (66%) compared to RYY (34%), and favored YTY (41.5%) over YCY (24.2%). In contrast, the frequency of CPD formation was lowest with a 5′-flanking G (GYY) (12%), and was particularly pronounced for TC sites, whereas it was 21.5% for YYG. On the 3′-side (YYN) there was no preference for a purine or pyrimidine (52% R versus 48% for Y). At the tetranucleotide level, CPD frequency was highest at YYYR sites (36%) and least for RYYY (18.5%) demonstrating a preference for CPD formation at the 3′-end of a polypyrimidine tract. The most favored CPD site was at TYYA, and these four sites out of 64 accounted for 12% of the CPDs compared to a statistical value of 6%. Analysis of NB UVB induced CPD formation in the 16 RYYYYR tracts (Figure 6), reveals that about half (9/16) form CPD’s preferentially at the 3′-end of the pyrimidine-tract. While a number of sites within the T-tract show enhanced CPD formation, the average frequency of CPD formation within the three dipyrimidine sites in an RYYYYR tract is only 1.7% compared to 1.0% for a RYYR site and a statistical probability of 1.6%. Flanking bases also had effects that were unique to particular dipyrimidine sites. The heat maps for NB UVB light show that TYYG and CYYT were unique hotspots for TT, and AYYT and TYYT were hotspots for CT and CC sites.

### Analysis of CPD formation by triplet photosensitization

**Figure 7.**
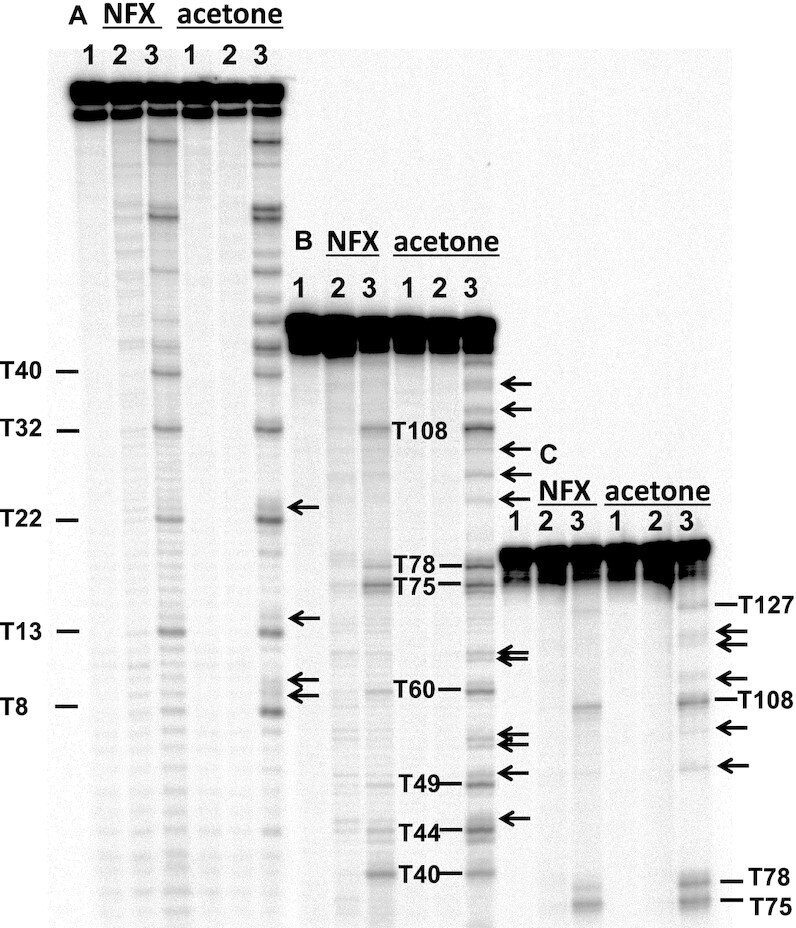
PAGE analysis of photosensitized CPD formation with norfloxacin (NFX) and acetone. The 5'-^32^P-labeled 149-mer was irradiated with 300 μM NFX/UVA light or 20% acetone/UVB light and digested with T4-pdg followed by hot piperidine, and sequentially loaded on a PAGE gel: (**A**) sample loaded last, (**B**) sample loaded second, and (**C**) sample loaded first. Lane 1: 149-mer treated with T4-pdg and hot piperidine; lane 2: 149-mer irradiated with UVA or UVB light in the absence of photosensitizer followed by sequential treatment with T4-pdg and hot piperidine; Lane 3: 149-mer irradiated with UVA or UVB light in the presence of photosensitizer followed by sequential treatment with T4-pdg and hot piperidine. CPD bands at 12 of the 16 possible NTTN sites are labeled according to the position of the 5′-T. Not labeled are sites 43, 74, 106 and 107. Arrows point to CPD bands containing C.

To investigate CPD formation by triplet photosensitization, the 149-mer was irradiated with NB UVB in the presence of acetone, which has a triplet energy higher than those of both T and C, or with UVA (320–410 nm centered at 365 nm) in the presence of norfloxacin (NFX) which has a triplet state that is barely higher than that of T. The concentration of the sensitizers and the length of UV irradiation time were adjusted to achieve similar levels of CPD formation under single hit conditions. The controls in this case consisted of DNA irradiated in the absence of photosensitizer and treated with T4-pdg and piperidine. Triplet sensitization with both acetone and NFX led to predominant formation of CPDs at TT sites, though acetone also lead to detectable CPDs at TC and CT sites (Figure 7). Because CPDs formed primarily at TT sites, they could be adequately resolved by sequential triple loading of the 149-mer. Exposure of DNA to NB UVB light for the same length of time, but in the absence of acetone, did not result in detectable formation of CPDs, indicating that all CPDs were being formed by photosensitization. Irradiation with UVA alone produced a number of bands with much lower intensity and with a different pattern that observed in the presence of NFX. With both sensitizers, we observed clearly resolvable cleavage bands for all possible XTTY sites. In accord with the high triplet energy of acetone, low but detectable CPDs were also found to form at some of the TC and CT sites, notably TTCC-9, TCCG-10, GCTA-19, TTCT-23, TTCA-45, TTCG-50, GCTC-53, CTCC-54, CCTC-64, CTCA-65, GTCC-90, CCTA-97, ACTA-103, GTCTCG-111-113, GTCA-116, ACTCTA-119–21 (indicated by arrows in Figure 7). These CPDs averaged to a total of 21 ± 1.5% of all quantifiable CPDs for three separate experiments, the remaining 79% being TT CPDs. In contrast, irradiation of the DNA with UVA light in the presence of NFX resulted in barely detectable CPD formation at C-containing sites compared to the control lane, and could not be quantified. Neither acetone or NFX produced any detectable CC CPDs, with the exception of TCCG-10 for acetone.

### Effect of flanking sequence on triplet sensitized CPD formation

**Figure 8.**
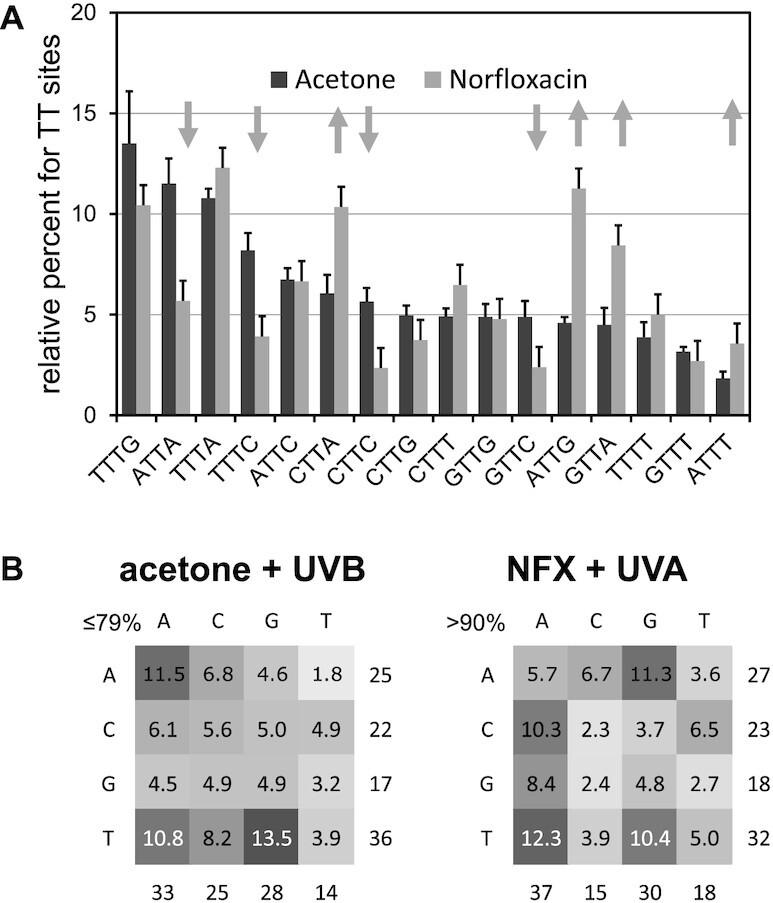
Sequence dependence of photosensitized CPD formation. (**A**) Barplot in decreasing percent of CPD formation at NTTN sites with acetone sensitization with arrows indicating >1.7-fold differences between the NFX and acetone sensitized CPD formation. (**B**) Heatmap of percent CPD formation at NTTN sites as a function of the photosensitizer. The labeling is the same as in Figure 5. The average and standard deviation for three experiments is shown in the barplot. Because CPD formation could only be quantified at a few C-containing sites in the acetone-sensitized reaction, the upper limit of photoproduct formation at TT sites is given, whereas none could be quantified in the NFX reaction, so only an estimated lower limit is given.

While the flanking sequence effects on both acetone and NFX sensitized CPD formation were similar to that for direct formation by UVC light (*R* ≈ 0.75), the flanking sequence effects were quite different between the two sensitizers (*R* = 0.47) (Table 1). Also, while acetone sensitized CPD formation showed similar correlation coefficients with all ranges of UV light (*R* ≈ 0.75), NFX sensitized CPD formation was most similar to UVC (*R* = 0.74) and less similar to UVB light (*R* ≈ 0.6). The similarities and differences between acetone and NFX sensitized CPD formation can be visually appreciated from the PAGE gel (Figure 7) and the bar graphs and heat maps (Figure 8). For both photosensitizers, relative CPD formation at TT sites was maximum at TTTR (average of 12%/site), but lowest at ATTT-106 (1.8%) for acetone and C/GTTC for NFX (2.3%/site). As was found for UV induction, CPD formation was favored at the 3′-end of the ATTTTG T-tract for both acetone and NFX photosensitization with CPD ratios for the first, second and third sites of 1.8:3.9:13.5 and 3.6:5.0:10.4, respectively. As also was observed for UV, photosensitized CPD formation by both acetone and NFX was lowest with a 5′-flanking G (17% and 18% respectively). NFX sensitized CPD formation was >2-fold less than for acetone at ATTA-78, TTTC-44, CTTC-49 and GTTC-8, and >1.7-fold greater than acetone at CTTA-32, ATTG-40, ATTT-106, and GTTA-2 sites.

## DISCUSSION

Over the years there has been great variation in the reported TT:TC:CT:CC CPD ratios for different wavelengths, doses and methods leading some to conclude that TT CPDs are the major product with UVC, and that its proportion decreases with increasing wavelength. One problem is that UVC is also capable of reversing CPDs (Figure 2A, path B) with a quantum yield close to 1 (73) and that 254 nm light is much better at reversing C-containing CPDs than TT CPDs due to their significantly greater absorbances at 254 nm (73,74). Therefore, if care is not taken to use extremely low fluences, the results can be biased by photoreversal (18,19). In our study, we used a dose of about 500 J/m^2^ of 254 nm light which appeared to be in the linear range for CPD formation, though some C-containing sites began to show curvature at higher doses (Supplementary Figure S6). Even so our TT:TC of 1.76 was much higher than a value of 1 observed in studies of naked DNA irradiated at much lower fluences of about 40 J/m^2^, suggesting that photoreversal was taking place. Our TT:TC ratio of 1.26 with 1.6 kJ/m^2^ of broadband UVB is also higher than about 1 observed in other studies using lower doses (25,27) or a study that filtered out light <290 nm (72), indicating that the lower UV wavelengths in our BB UVB were still causing some photoreversal. With NB UVB and filtBB UVB which lack the lower wavelengths, however, we observed a 1:1 TT:TC ratio and an overall ratio of 30:30:18:22 which is similar to the reported ratios for both low dose UVC and filtered UVB suggesting that the formation of CPDs in the UVC/UVB range is wavelength independent. A relatively invariant ratio of CPD formation at TT:TC:CT:CC was also found in a study of cells irradiated with low dose UVC, filtered UVB and simulated sunlight (70). Together, these results are consistent with the finding that the quantum yield of CPD formation in an oligothymidylate is independent of wavelength in the UVC/UVB range and that CPD formation results from a barrierless reaction involving a ^1^pi-pi* exciton (16). Thus, our NB and filtBB UVB data is expected to reflect the rate of UV-induced CPD formation in the absence of photoreversal and the NB UVB data will be used for the subsequent discussion of sequence effects.

Our data also indicates that (6-4) photoproduct formation does not compete with CPD formation (Figure 2A, path F) in spite of being formed with almost an equal frequency at TC sites with UVC but with diminishing frequency with increasing wavelength. It has been found by HPLC–MS/MS that (6-4) photoproduct formation occurs principally at TC sites with UVC light, and occurs with 82% of the efficiency of a CPD but with only 2% efficiency at CT sites, and that this drops to 65% and 0.8%, respectively, with BB UVB (25). In a more recent study, the ratio of (6-4)/CPD formation was found to decrease by a factor of about 5 on going from UVC (254 nm) to UVB (315 nm) (75). The 5-fold decrease in (6-4) photoproduct yield is consistent with the ∼4.5-fold drop in the ratio of the quantum yields for (6-4) and CPD formation from 254 to 310 nm that was attributed to a charge transfer mechanism with an energy barrier (16). If there was competition for the same excited state, one would therefore expect that the ratio of TC:CT CPDs would greatly increase with increasing wavelength. Our finding that the TC/CT CPD ratio is about 1.5 for all UV sources including UVC (254 nm) and NB UVB (311 nm), together with the in vivo results showing essentially the same thing (70) suggests that this competition does not play a role.

While the average CPD yields for TT, TC, CT and CC sites vary by about 1.7 for NB UVB, the CPD frequencies within a given dipyrimidine vary about 6-fold with flanking sequence, and 12-fold for all 64 sites. Hotspots for CPD formation were observed at the 3′-end of RYYYYR tracts, which has been previously observed with T-tracts (76,77). The preference for CPD formation at the 3′-end of a T-tract has been proposed to arise from the unique conformation of a T-tract, which involves a narrowed minor groove, increased propeller twisting and A-stacking which reduces the flexibility of all but the 3′-most TT site (76). The preference for the 3′-end was also observed for triplet sensitization, as we have observed for ATTTTG-106–108 indicating that the effect does not depend on the multiplicity of the excited state or that a common excited state is involved. Half of the 16 RYYYYR pyrimidine tracts in our sequence showed preference for CPD formation at the 3′-end of the pyrimidine tract (Figure 6), indicating that the unique conformation of T-tracts is not required for this effect. There was a clear preference for YYTY sites, with the exception of CCTT, and was not favored at YYCY sites. This contrasts with the reported effect of C on CPD formation in a T-tract which results in a dramatic reduction in CPD formation where the replacement is made, and an enhancement of CPD formation at the 5′-flanking TT (76). Given the variety of RYYYYR tracts that favor CPD formation at the 3′-end it seems unlikely that a specific conformation is involved, but it may well have to do with increased flexibility of the pyrimidine at the end of the tract when it abuts the purine combined with other conformational and electronic effects. In two cases, the ordering was reversed with the most favored site at the 5′-end as seen for ATCCTG, and ACCTTG. While a number of sites within the pyrimidine tracts show enhanced CPD formation, the average frequency of CPD formation within the an RYYYYR tract is only 1.7% compared to a statistical probability of 1.6% indicating that there is nothing intrinsically special about polypyrimidine tracts.

As suggested by previous studies with limited sequence coverage (4,18,30,33,35,36), the frequency of CPD formation by UVB light was uniformly lowest for a dipyrimidine site when flanked by a 5′-G, GYY (9–15%), and was particularly pronounced for TC sites (9%) (Figure 5). The suppression of CPD formation by a 5′-G is consistent with the proposal that the excited state is quenched by electron transfer from a G to an excited T to form a charge transfer complex (Figure 2A, path G), and that a 5′-G is better able than a 3′-G to quench the excited state because it has greater pi overlap (4,35–37). A theoretical study on GTT, GTC, GCT and GCC, however, concluded that a 5′-G would have a more significant effect on quenching the reactivity of CPD formation when flanking a T as in GTT and GTC than a C in GCC and GCT (37), which is not what we observed. Furthermore, it was concluded that the next flanking base would also have a lesser effect, but would favor quenching at GTT more than GTC, which is also not what we observed. Interestingly, among the four dipyrimidine sites, CT also showed equal inhibition of CPD formation when flanked by a 3′-G, largely as the result of low CPD formation at TYYG, which is otherwise favored by all the other dipyrimidines. The ability of a 5′-G to inhibit CPD formation was also observed for triplet sensitized CPD formation at TT by both acetone and NFX (17 and 18%, respectively, for GTT vs 28 and 30% for TTG), suggesting that charge transfer quenching may also operate for lower energy triplet excited states.

In comparison to direct formation of CPDs by UV irradiation, the relative yield of TT versus C-containing CPDs formed by photosensitization with acetone (≤79%) and NFX (>90%) was much higher than that for NB UVB (31%), and consistent with previous studies with sensitizers (41,53,56). The low fraction of C-containing CPDs has traditionally been explained by the fact that T has a lower triplet energy than C, and that triplet states will either preferentially form at, or migrate to, a T. This difference alone, however, would not readily explain why CPDs can’t also form at TC and CT sites which also contain a T. Recent time-resolved studies of CPD formation upon triplet sensitization of T in TT, TC and CT found that the quantum yield is 2–3 less for TC and CT formation than for TT formation, presumably reflecting the relative stability of the biradical intermediates (Figure 2B) (51). While this would explain why TT CPDs are formed in higher yield than TC and CT CPDs, it does not explain why the TT/(TC + CT) ratio depends on the triplet energy of the sensitizer, unless the reaction can also proceed through the triplet state of C. Acetone has a triplet state energy of ≈340 kJ/mol (52) that is higher than that for both T and C, whereas NFX has a much lower energy triplet of ≈270 kJ/mol that can only sensitize T (46,49). One interesting proposal to explain the triplet energy dependence, is that triplet sensitized CPD formation in DNA involves simultaneous single electron transfer to one base, and back electron transfer from the other base to form a radical ion pair, rather than to and from a single base (41). If the oxidation potential of one of the bases is too high such as C, CPD formation would be inhibited.

Another possible mechanism that might contribute to the lower frequency of CPDs formed at TC and CT sites that might also explain the significantly different flanking sequence effects between NFX and acetone (arrows in Figure 8), is sequence specific static quenching of the excited singlet state of NFX by DNA. Acetone is a small molecule with no particular binding affinity for DNA, whereas NFX is a zwitterionic, flat aromatic molecule, that has been found to intercalate DNA based on NMR and linear dichroism studies (78), with a preference for GC rich sites (79). As further evidence of intercalation, the triplet energy of NFX has been reported to increase to 299 kJ/mol upon binding to DNA (48). Triplet energy transfer by acetone is therefore expected to occur by a dynamic mechanism involving random collisions (Figure 2B, Path A) whereas NFX may be able to transfer triplet energy via pi stacking with a pyrimidine base in an intercalated complex (Figure 2B Path D). One might therefore expect that triplet sensitized formation of CPDs by NFX would reflect the sequence specific binding of NFX to DNA and favor GC-containing dipyrimidines sites, unless it were to intercalate between the pyrimidines, and thereby prevent CPD formation. While NFX may have an intrinsic preference for binding to GC rich sites, its singlet state has also be shown to be quenched by a static process involving electron transfer from DNA which would diminish the triplet yield (80). Evidence that fluorescence quenching is occurring by electron transfer from G (Figure 2B, Path E) comes from the observation that 8-oxo-dG is formed in competition with CPDs upon photosensitization with NFX (81), though it might also have arisen via a singlet oxygen pathway (82). Our studies were carried out with nitrogen purged samples which should have suppressed this pathway.

Fluorescence quenching by G has recently been proposed to explain why the triplet states of psoralen are not detected in heterogenous DNA (83,84) and might explain why hotspots for photosensitized formation of CPDs by the intercalator pyridopsoralen which has an almost identical triplet state energy of 273 kJ to that of NFX (85) were observed to occur primarily at AT-rich sequences (57). The five hotspots for NFX sensitized CPD formation were also observed at AT-rich sites ATTG, CTTA, GTTA, TTTA and TTTG, the first three of which were unique for NFX, while the latter two were also observed with acetone sensitization and direct irradiation. Interestingly, of these sites, TTTA and CTTA were also found to be hotspots for pyridopsoralen (57). CPD formation by NFX was inhibited by a 5′-G, as was observed for acetone sensitization, but this could be due to charge transfer quenching of the triplet excited pyrimidine as we observed for CPD formation by direct excitation with UV light. NFX sensitized CPD formation was also inhibited, however, by a 3′-C, and was two-fold lower at TTTC, CTTC and GTTC than for acetone which could be due to charge transfer quenching of NFX from the G pairing to the 3′-C. Further complicating the interpretation of sequence effects is evidence that charge transfer quenching by G may also take place through A’s (86,87), and that triplet-triplet energy transfer can occur over several base pairs (42). It is likely then that the sequence specific difference between NFX and acetone sensitization arise from a complex competition between static quenching and triplet-triplet energy transfer that depends on the orientation and pi stacking geometry of the NFX relative to the dipyrimidine site.

While there are some sequence-specific differences between direct and triplet sensitization of CPD formation at TT sites that can be attributed to the properties of the sensitizer, the similarity of the sequence contexts effects such as suppression of TT CPD formation by a 5′-G, and a preference for CPD formation at the end a of T-tract TTTTR is curious. One mechanism proceeds through direct absorption of light followed by a concerted mechanism that takes place in a picosecond, while the other proceeds through a physical interaction with an excited triplet molecule, leading to a biradical intermediate that subsequently has to ring close to form the CPD (50,51). One explanation would be that both direct and sensitized CPD formation proceeds through the same excited state, which if they did, would have to be the lower energy triplet state. Some early work did propose the involvement of the triplet state in CPD formation following direct irradiation but was followed by another study that concluded that the triplet state is not involved (88,89). If the latter is the case, it would have to be that the concerted and stepwise mechanisms are subject to similar sequence dependent excited state localization and conformational effects on dimerization.

Given our observations and those of others that both acetone and NFX sensitize CPD preferentially at TT sites, it is difficult to understand the relative frequency of CPD formation at TC and CT sites reported to occur in the dark by a chemisensitized pathway following UV irradiation of melanocytes (9). In the proposed pathway, a high energy molecule is produced through an oxidative process that decomposes to a triplet excited state which then photosensitizes CPD formation in DNA. A ratio of 0.37 was reported for (TC + CT)/TT CPDs immediately following irradiation with UVA light, which corresponds to 27% TC + CT (assuming that no CC CPDs are formed), which is much greater than the 13% previously reported for UVA irradiated CHO cells (55). What is more surprising and not consistent with our results or those of others with triplet sensitizers, is that in the 2 h dark period, the (TC+CT)/TT CPD ratio was reported to rise from 0.37 to 1.3 which corresponds to 57% TC + CT. The ratio of 57% induced by the chemisensitized pathway is much higher than the 21% that we observed with acetone, which has the highest triplet energy (337 kJ/mol) of carbonyl compounds that have been studied as DNA photosensitizers. Lower energy triplet sensitizers give equal or lower fractions of TC+CT, such as 20% for acetophenone (317 kJ/mol) and 12% for norfloxacin (≈270 kJ/mol) (41). Norfloxacin has the lowest known energy capable of photosensitizing CPDs in DNA (46,49), and did not produce quantifiable TC+CT CPDs in our study. If the dark CPD ratios are accurate, it would suggest that the aromatic carbonyl compound (2-oxalylamido,4,5-dihydroxybenzaldehyde) that was proposed as a possible triplet sensitizer arising from thermal decomposition of the 2,3-dioxetane of dihydroxyindolecarboxylic acid (DHICA) (9) is unlikely to be the sensitizer. Such a compound would be expected to have a triplet energy that is similar to that of the closely related carbonyl compound N-formylkynurenine, NFKU, and would not be expected to produce the large fraction of CPDs at TC and CT sites found in the dark pathway due to its low triplet energy of 272 kJ/mol (90). The only way to reconcile the reported data is that either the fractions of TC + CT were overestimated, or that the chemisensitizers involved have a high specificity for TC and CT sites, and/or have triplet state energies that are much higher than that of acetone.

## CONCLUSION

In summary, we have designed a sequence that allows for a comprehensive comparison of the flanking sequence context effects on direct and photosensitized formation of CPDs which has revealed differences due to the exciting wavelength, and the structure and properties of the triplet sensitizer. Such a sequence could also be used for studies of sequence context effects of C5 methylation on CPD photoreversibility, (6-4) photoproduct formation and isomerization to the Dewar product, deamination of CPDs, as well as for CPD formation by other photosensitizers and chemisensitizers. It could also be used to study sequence contexts effects on repair of CPDs by glycosylases, photolyases, and excinucleases. While it is likely that sequence context beyond the immediately flanking bases can influence CPD yield, the sequence context effects discovered for direct and photosensitized CPD formation in our sequence could be used to guide comparative theoretical calculations of CPD formation as a function of flanking sequence, spin state and photosensitizer. Such studies might help determine whether the flanking sequence effects are due to localization of the excited states, and/or to altering the geometry and the dynamics of the reacting bases and sensitizers. Further experimental and experimental studies with triplet sensitizers of different energy and structure may also help to narrow down the types of chemisensitizers involved in CPD formation by the dark pathway in melanocytes.

## DATA AVAILABILITY

The data and source code for the software used to generate the sequences are available at https://github.com/lvchen727/149mer-paper.

## Supplementary Material

gkab214_Supplemental_File
