# Supplementary material for: The effect of flanking bases on direct and triplet sensitized cyclobutane pyrimidine dimer formation in DNA depends on the dipyrimidine, wavelength and the photosensitizer
Source: Nucleic Acids Res. 2021 Apr 13;49(8):4266–80. doi: 10.1093/nar/gkab214 (PMC8096240; doi:10.1093/nar/gkab214)
Supplement: gkab214_Supplemental_File [file gkab214_supplemental_file.pdf]

The effect of flanking bases on direct and triplet sensitized cyclobutane pyrimidine dimer formation in DNA depends on the dipyrimidine, wavelength, and the photosensitizer

Chen Lu, Natalia Eugenia Gutierrez-Bayona and John-Stephen Taylor*

Department of Chemistry, Washington University, One Brookings Dr., St. Louis, MO 63130, USA

** To whom correspondence should be addressed: Tel: +1-314-935-6721. Email:* taylor@wustl.edu

Figure	Page
Figure S1. Examples of other 129-mer sequences containing one each of all 64 possible NPyPyN sites.	2
Figure S2. Sequences used to assemble a 171-mer single strand containing the 149-mer sequence by template-directed ligation.	3
Figure S3. Sequence analysis of the 149-mer containing clone.	4
Figure S4. Absorbance and emission spectra.	5
Figure S5. Time dependence of CPD induction by UVC light.	6
Figure S6. Quantitative analysis of the time dependence of CPD induction by UVC light.	7
Figure S7. T4 pdg concentration study and effect of 5 min of 1 M piperidine 90°C on BB UVB irradiated DNA.	8
Figure S7. T4 pdg concentration study and effect of 5 min of 1 M piperidine 90°C on BB UVB irradiated DNA.	9
Figure S8. Standard deviation of the average percent cleavage at various CPD sites in the 149-mer sequence for a 20-fold range of T4 pdg concentrations.	10
Figure S9. Hot piperidine cleavage of the 79-mer with and without prior UVC irradiation or T4-pdg treatment.	11
Figure S10. Barplots of the average frequency of CPD formation for UV irradiation as a function of sequence context.	12-13

Supporting Figure S1.

DNA1:
GTTCCATTCTACTTCGTCTTGCCATTACCGCTATCCTGTTGCTTTGCCTTATCACTCTGCTCC
GTCACTGCCGTCGTCCCACCCTACCATCTCATCGCCCCGTTACTATTGCTGTTTATTTCACC
TCG
DNA2:
GTTCCATTATCCCGTCGTTTTGCTGCCACCCCATTCTGCCGCTATCTCATTGTCCTATCGTCA
TTTCACTTTACTCTACCACCGCCTCGTTGCCCTGCTCCGTCTTGTTACCTTATCACTACTGCT
TCG
DNA3:
ACCTTATCTCACTTCGCTACTGTCACCATTATCCTACTCTGCTTTGTCGTTTTATCGCCGTTA
CCCTGTTGCCATCACTATTGTCTTGCCCCACCGCTCCGTCCCGCCTCGCTGTTCTATTTCATT
CCA
DNA4:
ACCGCTTTGCCTCATCCTGTTGTTTTACCCCGTTATTTCGCCGTCATCGTCTCGTCGCTCCGC
CCTACTTCACTGCCATTATTGCTGTTCTGTCCCATCTTGCTATCATTCCACCACTCTACTACC
TTA
DNA5:
ACCCCATCCTGCCCTATTCCACTTCATCTCGCTATTGTTGTCATTTTACCTCATCACCGTCTT
GCTGTCCCGCCGTTTCGCTTTGCCTTATTACTACCATCGCTCCGTCGTTCTGTTACTGCCACT
CTA
DNA6:
GCTGTCGTTCCGTTATCGCTATTCTGCCATCTTACCCCATTGTTTCACTTCGCTCCACCTTGC
CTCATCCTGCTTTATTACCACTGCCGTCCCGCCCTATCACTATTTTGTCTCGTCACTCTACCG
TTG
DNA7:
GTTGTCATTGCCGCCTCGCTGTTTTGCCACCGTCTCACTACCACTCCATCATCCCACTTCGC
CCTGCTCTATTTCACCTTGCTATTATCTTATCGTTACTGTTCTGCTTTATTCCGTCCTACCCCG
TCG
DNA8:ACTATTTCACTTCGCTTTGTCTCGTTGTCATTCCGTTTTACTCTGCCGCTCCATTGCCT
CACCGTTCTACTGTCGTTATCTTATCGCCACCTTGCCCTGTCCTATTACCCCGCTGCTATCCC
ATCACCA
DNA9:
GTCACCGTCCCGCTCTATTCTGCTTTGTTTCGCTATCTTATTGCCCCACTGCTGTTACT
CCGTCTCATCCTACTTCATCGCCTTGTTGCCGTTCCACCACCCTGCCATTTTATTACTA
TCACCTCGTCG

Figure S1. Examples of other 129-mer sequences containing one each of all 64 possible NPyPyN sites. DNA9 was the sequence studied in this paper.

Supporting Figure S2.

Strand 1: (48-mer)
GCATCGAATTCGTGCACGTGCGCTGTCGTTCCGTTATCGCTATTCTG
C

Strand 2: (42-mer)
pCATCTTACCCCATTGTTTCACTTCGCTCCACCTTGCCTCATC

Strand 3: (41-mer)
pCTGCTTTATTACCACTGCCGTCCCGCCCTATCACTATTTTG

Strand 4: (40-mer)
pTCTCGTCACTCTACCGTTGCACGCGTACAGAATTCGATGC

Template 1-2: (39-mer)
GTGAAACAATGGGGTAAGATGGCAGAATAGCGATAACGG

Template 2-3: (34-mer)
GGCAGTGGTAATAAAGCAGGATGAGGCAAGGTGG

Template 3-4 :(35-mer)
CGGTAGAGTGACGAGACAAAATAGTGATAGGGCGG

Figure S2. Sequences used to assemble a 171-mer single strand containing the 149-mer sequence by template-directed ligation. The EcoRI sites are underlined. All sequences are written 5'-3'.

Supporting Figure S3.

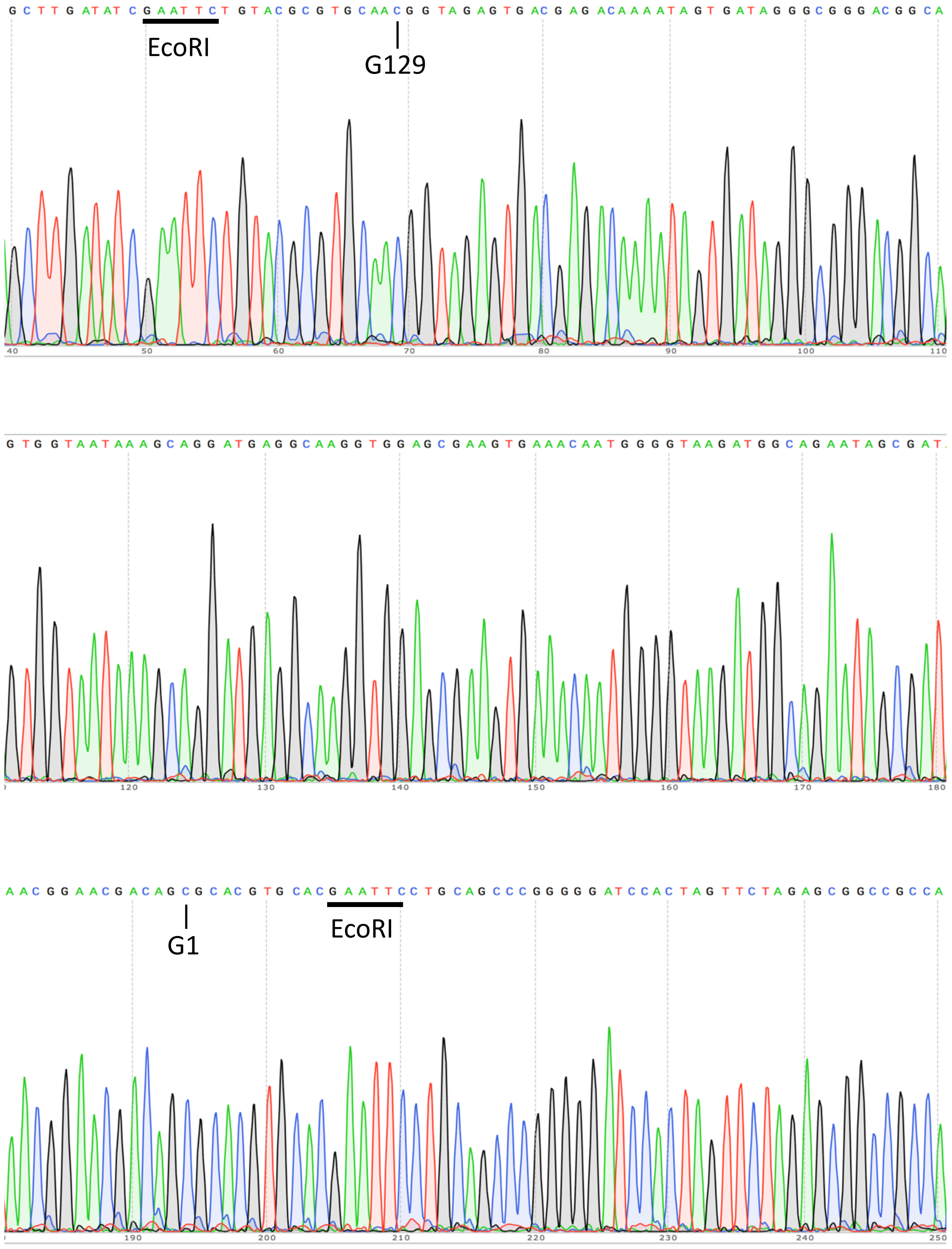

Figure S3. Sequence analysis of the 149-mer containing clone. The sequence shown is that of the strand complementary to the dipyrimidine containing strand. The two EcoRI sites and G1 and G129 of the 129-mer dipyrimidine library containing sequence are shown.

Supporting Figure S4

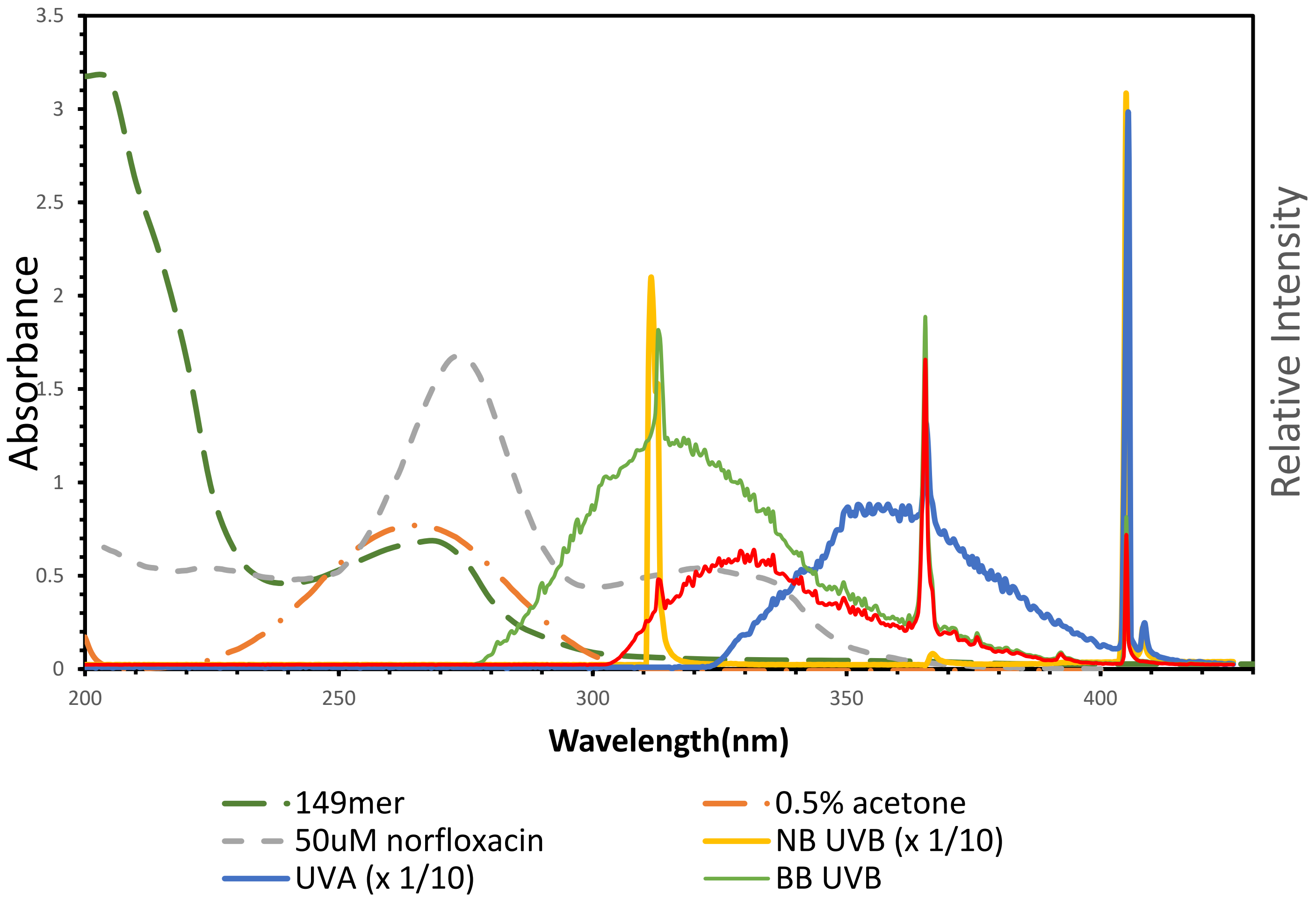

Figure S4. Absorbance and emission spectra. The dashed lines are the absorbance spectra of the photosensitizers and the 149-mer duplex. The solid lines show the relative intensity of the light produced from the UV lamps and in combination with a filter.

Supporting Figure S5

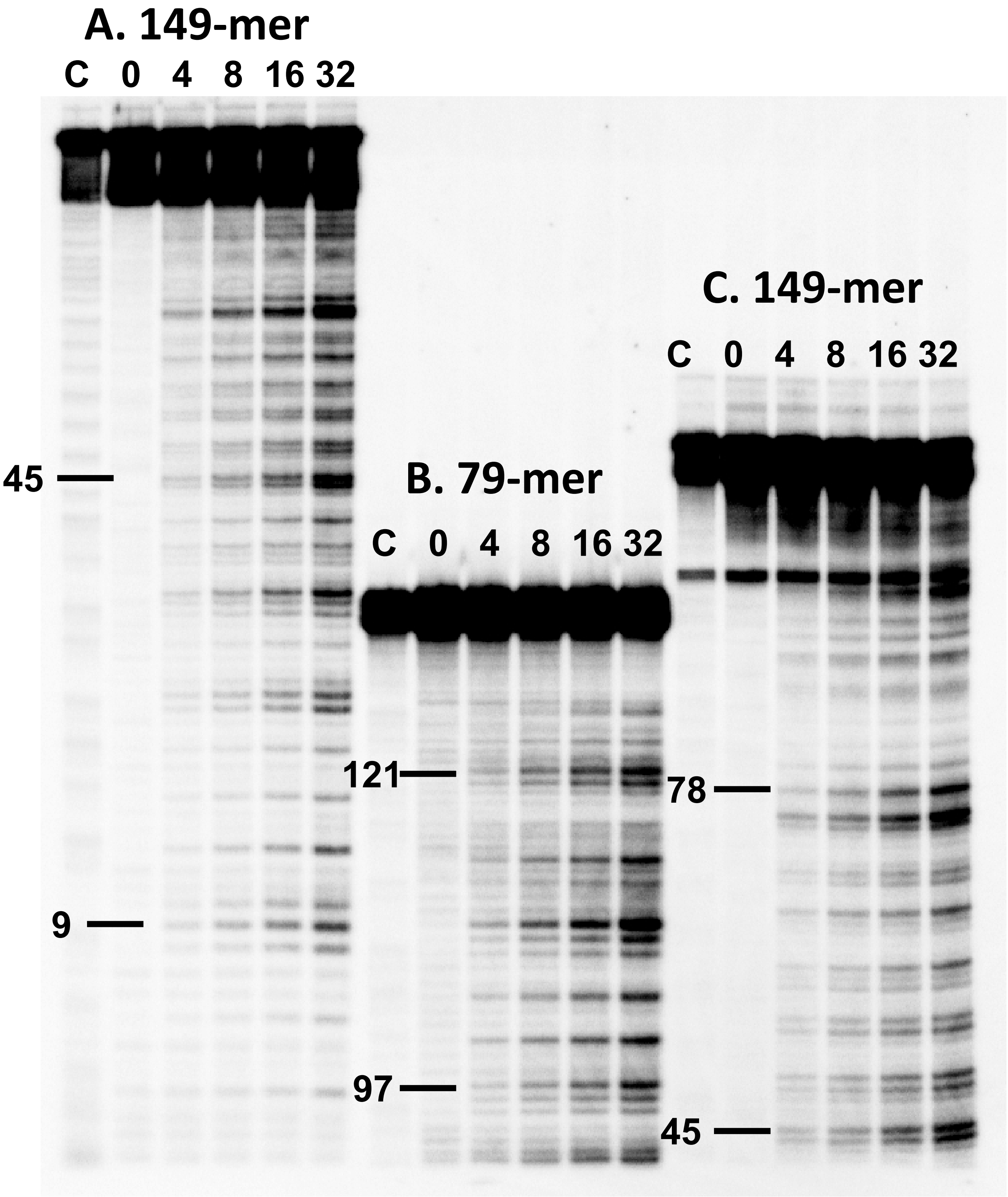

Figure S5. Time dependence of CPD induction by UVC light. The 5'-radiolabeled sequences were exposed to UVC light for the indicated times in seconds, and then cleaved with T4 pdg followed by piperidine. The numbers refer to the position of the bands in the sequence.

Supporting Figure S6

A.

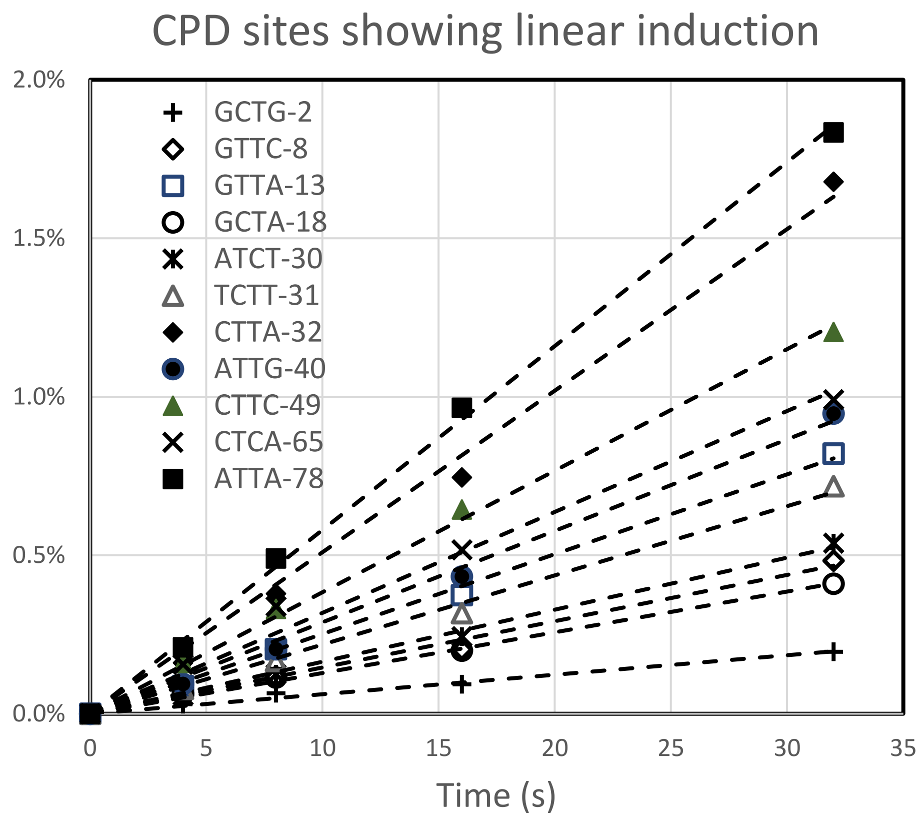

B.

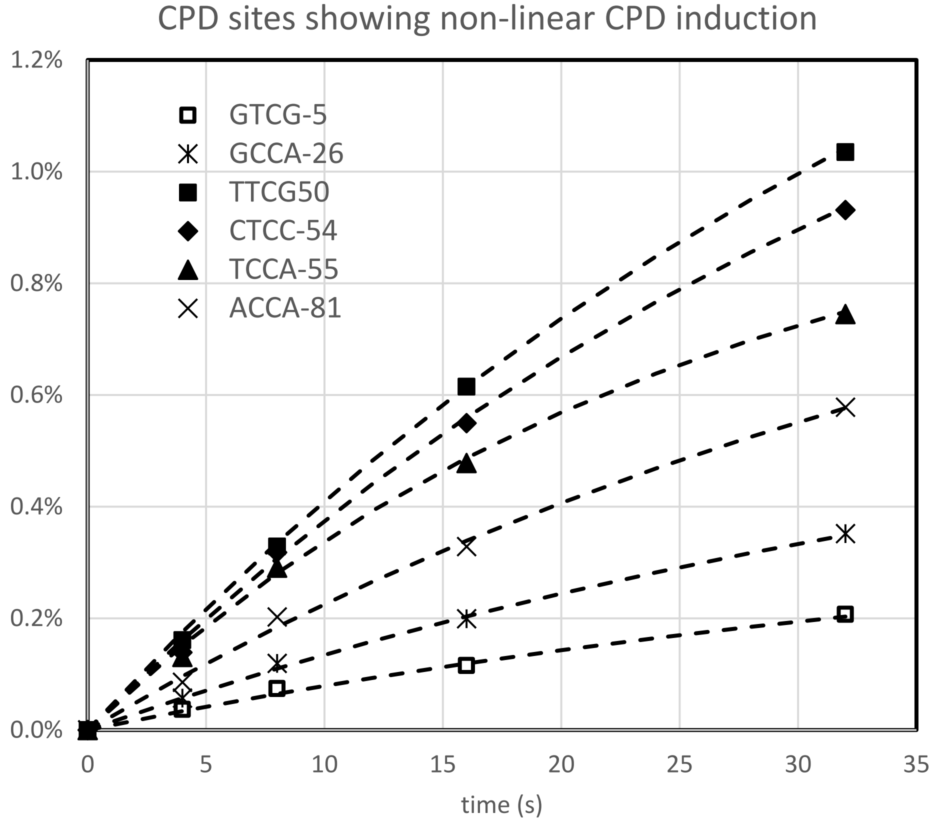

Figure S6. Quantitative analysis of the time dependence of CPD induction by UVC light. The 149-mer lanes in the gel shown in Fig. S5 were quantified by a phosphoimager and analyzed according to Poisson statistics to give the frequency of CPD formation as a function of exposure time. The sites in panel A showed linear induction over 32 s (approximately 2 $kJ/m^{2)}$, whereas the sites in panel B appeared curved and better fit an exponential growth curve,.

Supporting Figure S7

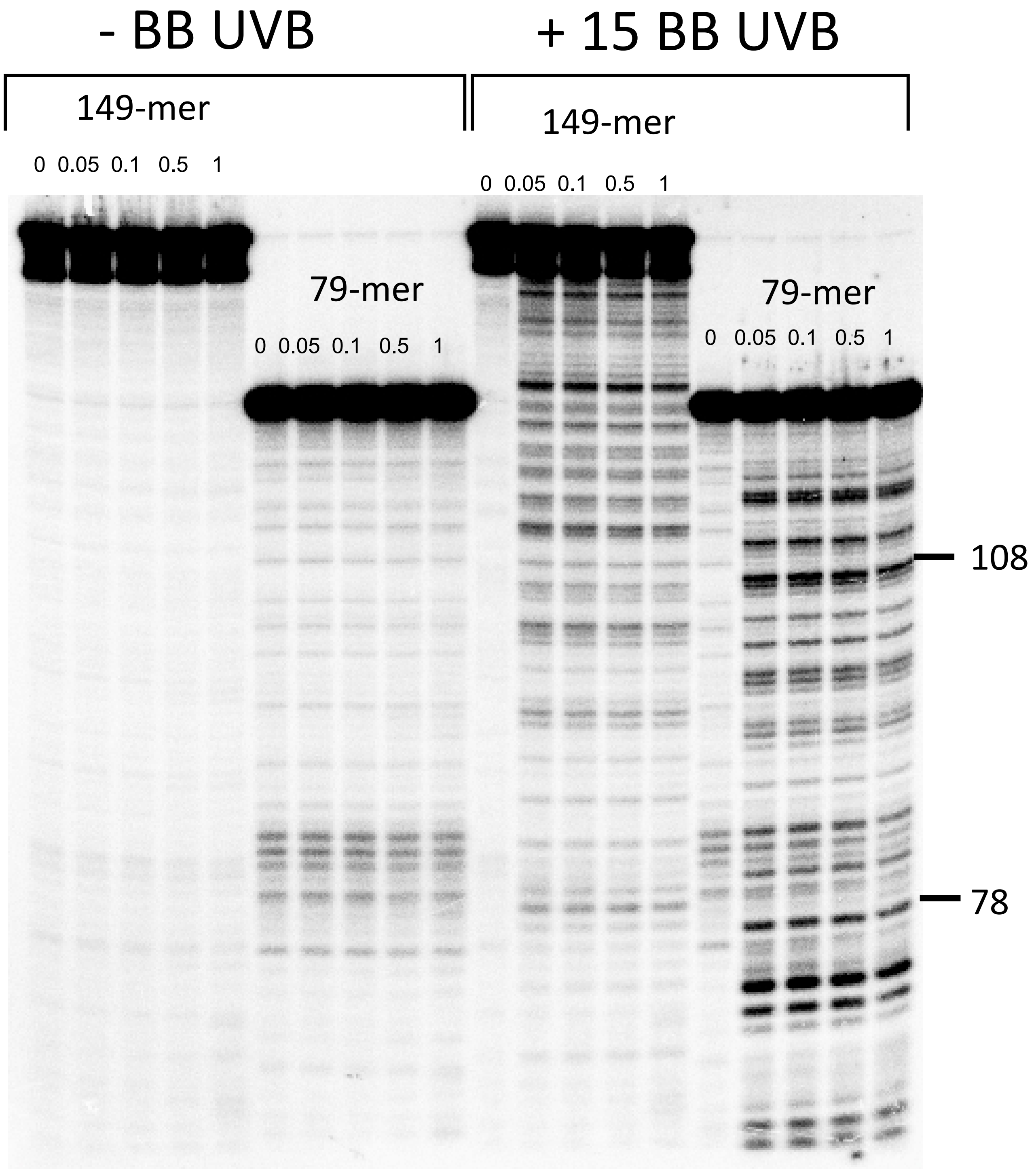

Figure S7. T4 pdg concentration study and effect of 5 min of 1 M piperidine 90°C on BB UVB irradiated DNA. DNA (+/- 15 s BB UVB) was treated with the indicated number of micrograms of T4 pdg in a 20 uL reaction volume followed by 5 min of 1 M piperidine at 90 °C. The concentrations in ug/20 uL correspond to 0.16 uM, 0.32 uM, 1.6 uM, and 3.2 uM. The percent background cleavage in the 149-mer resulting from 5 min treatment with 1 M piperidine at 90°C only increased by 8% upon prior exposure to 15 s of BB UVB light. The 79-mer had substantial background bands and was not analyzed.

Supporting Figure S8

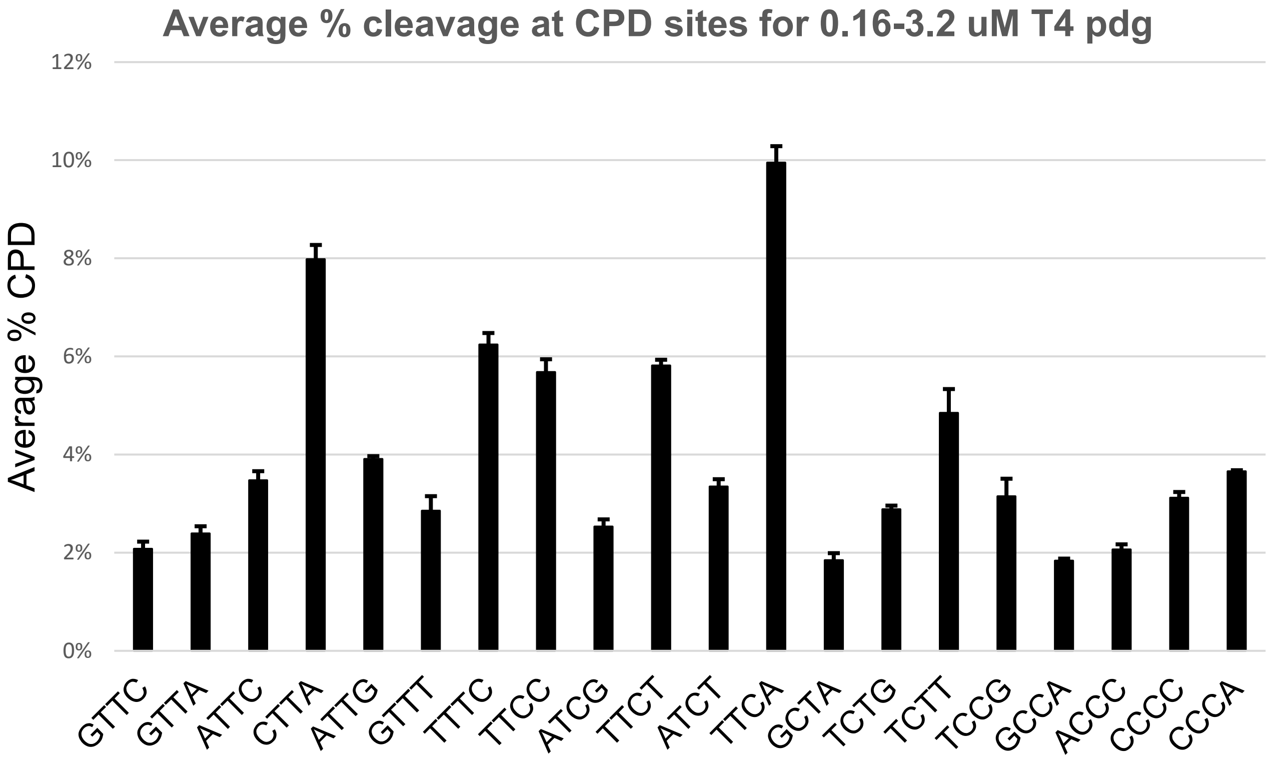

Figure S8. Standard deviation of the average percent cleavage at various CPD sites in the 149-mer sequence for a 20-fold range of T4 pdg concentrations. The T4 cleavage bands in Figure S7 for each of the four different T4 pdg concentrations (0.16, 0.32, 1.6, & 3.2 uM) were quantified and converted to percent cleavage by Poisson analysis as described in the main text. The percent cleavage at TT, TC, CT and CC sites in different sequence contexts for the different T4 pdg concentrations were then averaged and plotted as a bar showing the standard deviation.

Supporting Figure S9

G Reaction

		X	X	X	X		10 s NB UVC
			X				UVB 30 min
	X			X			T4 pdg
X	X	X	X	X			Hot pip 5 min

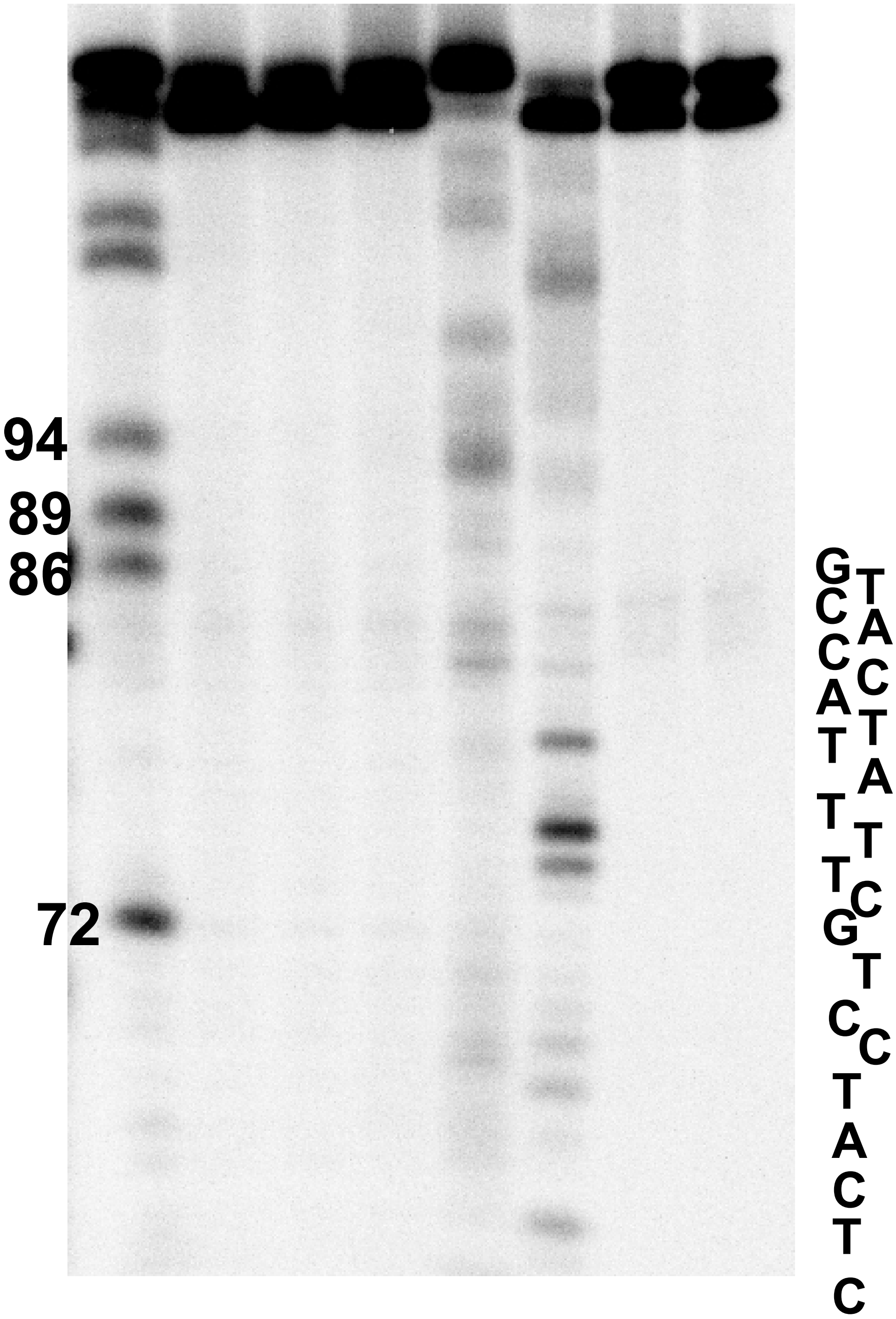

Figure S9. Hot piperidine cleavage of the 79-mer with and without prior UVC irradiation or T4-pdg treatment. The 79-mer PCR product was treated as given in the table above the gel sequentially from top to bottom. The percent background cleavage for treatment with 90 ^{o}C 1 M piperidine only increased by 10% upon prior exposure to 10 s of NB UVC light.

Supporting Figure S10 A&B

A.

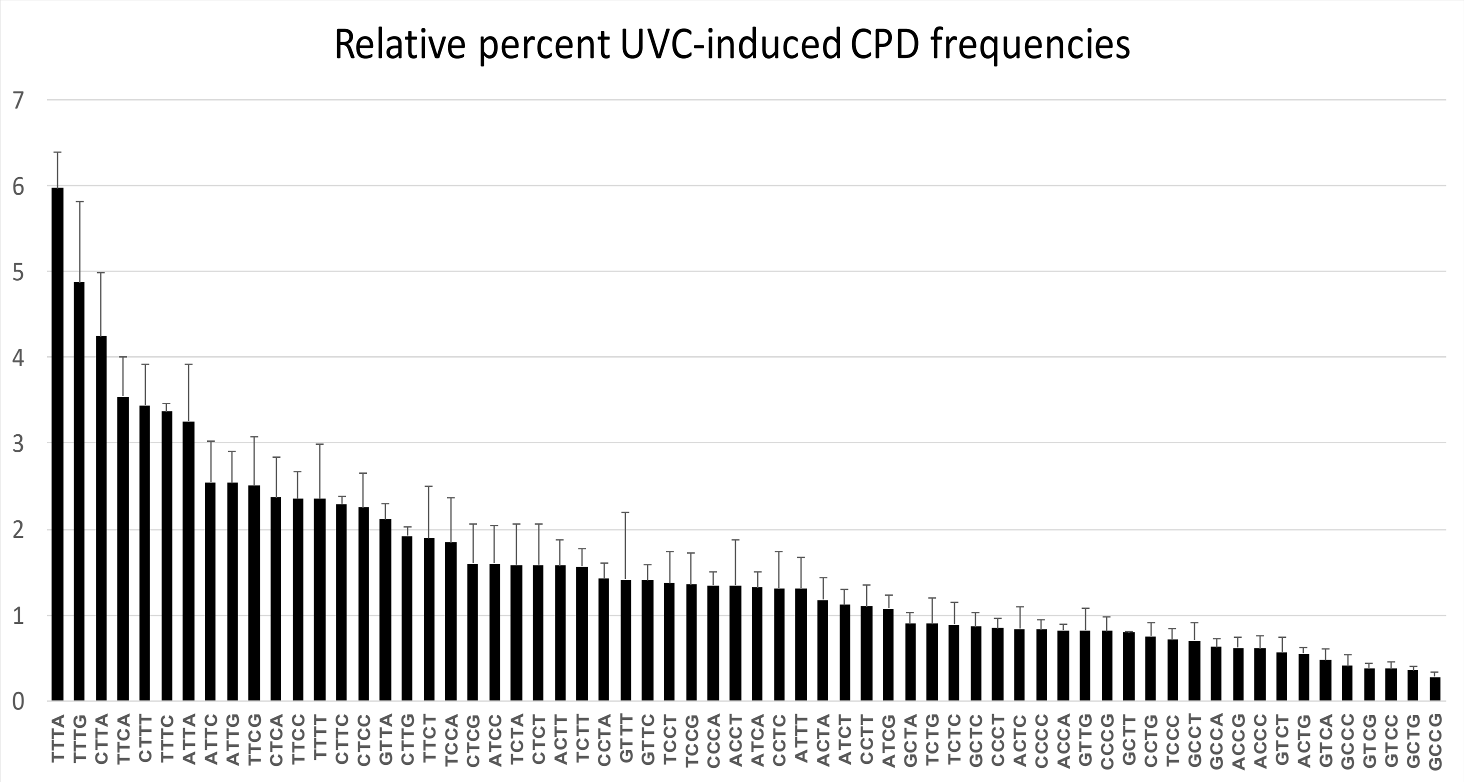

B.

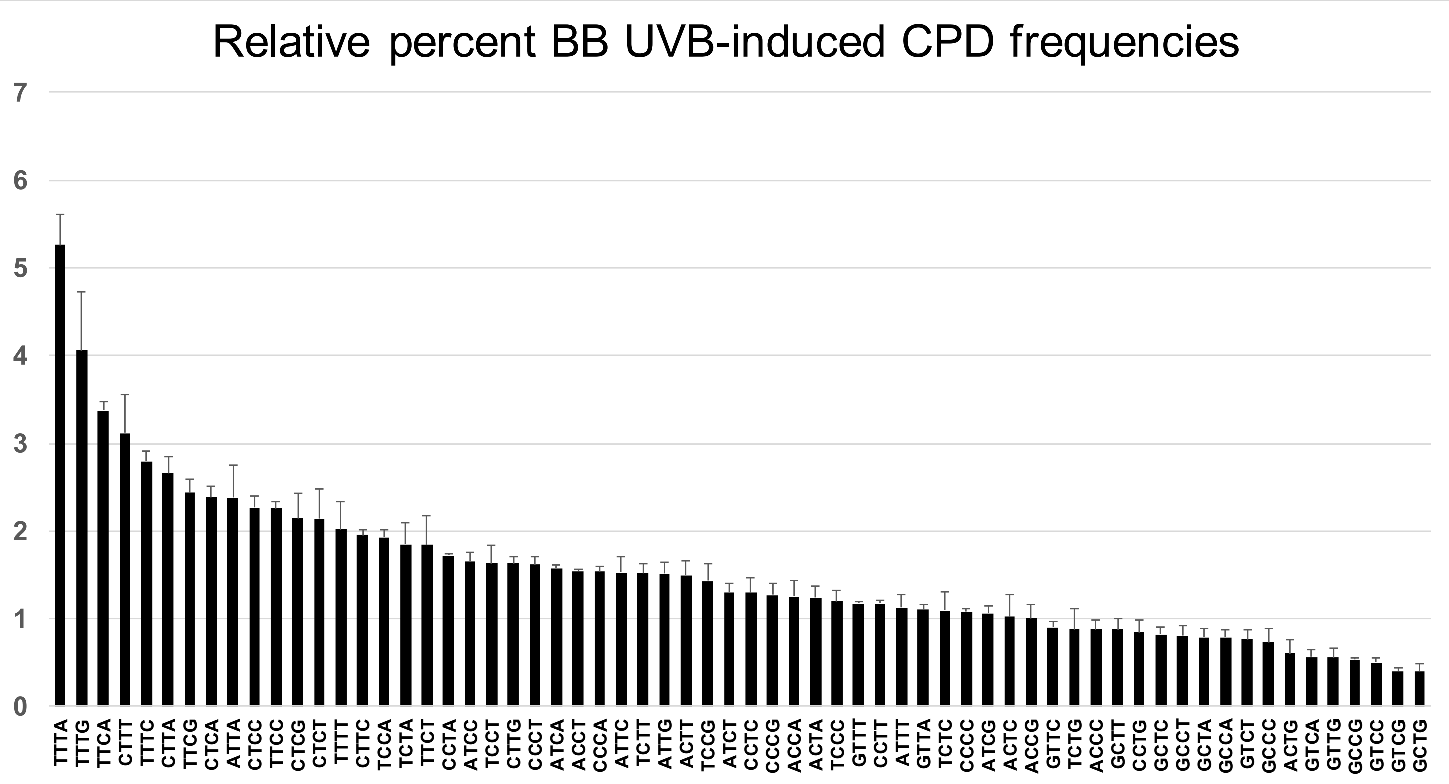

Supporting Figure S10 C& D

C.

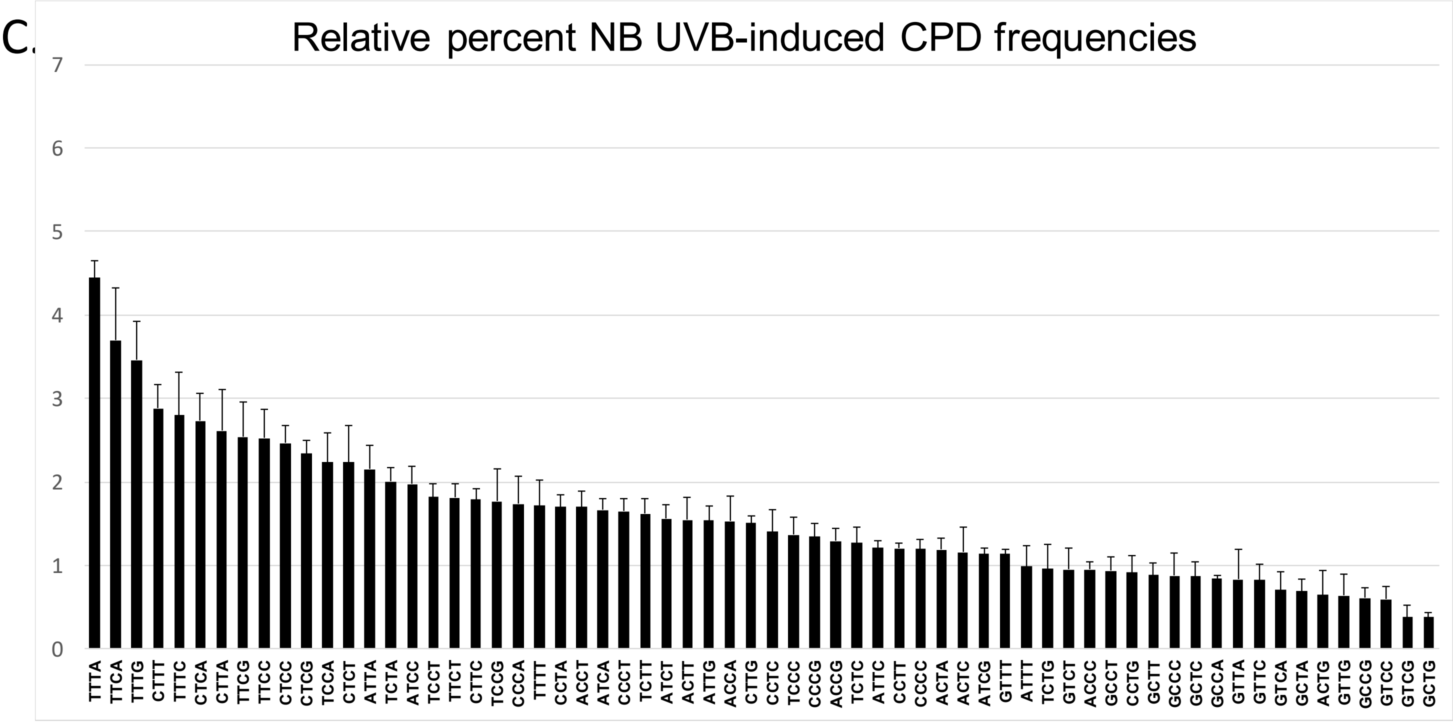

D.

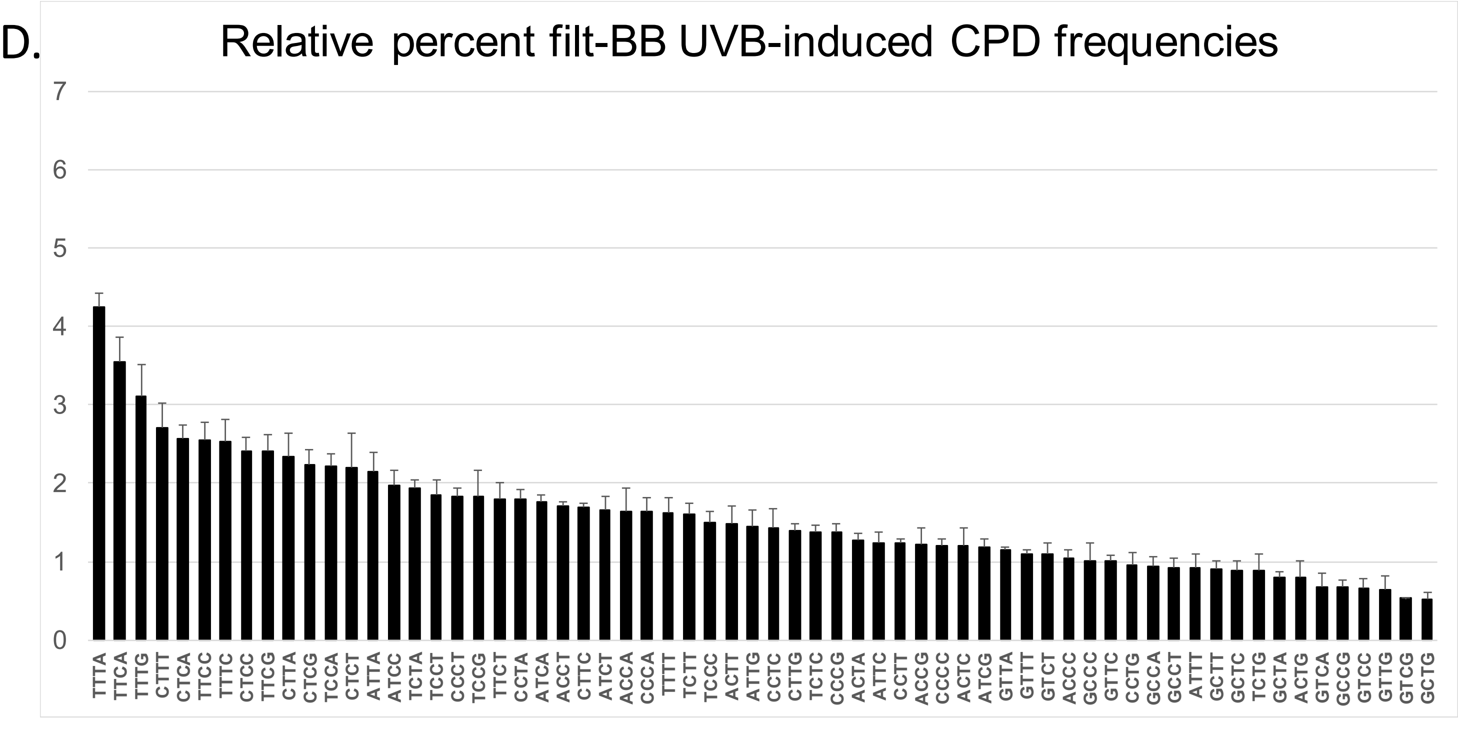

Figure S10. Barplots of the average frequency of CPD formation for UV irradiation as a function of sequence context. The sequences are ranked according to decreasing average frequency of CPD formation. The average and standard deviation is shown for separate three experiments.
